# Global solutions for stochastically controlled fluid dynamics models

**DOI:** 10.1007/s40072-025-00396-7

**Published:** 2025-10-10

**Authors:** Oana Lang, Dan Crisan

**Affiliations:** 1 Department of Mathematics, Babeş-Bolyai University, Cluj-Napoca, Romania; 2 Department of Mathematics, Imperial College London, London, UK

**Keywords:** Stochastic partial differential equations, Stochastic control, Fluid dynamics models, Global solutions

## Abstract

For a class of evolution equations that possibly have only local in time solutions, we introduce a stochastic component that ensures that the solutions of the corresponding stochasticallys perturbed equations are global in time. The class of partial differential equations amenable for this type of treatment includes the 3D Navier-Stokes equation, the rotating shallow water equation (viscous and inviscid), 3D Euler equation (in vorticity form), 2D Burgers’ equation and many other fluid dynamics models.

## Introduction

A large class of partial differential equations (PDEs) exists in the literature for which global existence of solutions does not necessarily hold, with several such examples coming from geophysical fluid dynamics, see e.g. [5, 8, 19, 20]. In this paper, we show that the addition of an appropriate stochastic term to this type of PDEs ensures existence of a global solution. The stochastic term can be chosen to act on the solution selectively: for example, it can act only when the solution of the PDE becomes too large in a suitably chosen norm. The class of PDEs that can be considered for this treatment has the form

1.1 $$\begin{aligned} dX_t = A_t(X_t)dt \end{aligned}$$

where *A* is a (possibly) time dependent nonlinear operator. We use the differential form of the PDE as this enables us to naturally extend the equation to its stochastic version. A representative example of such a PDE is the 3D vorticity equation

1.2 $$\begin{aligned} d \boldsymbol{\omega }+ u\cdot \nabla \boldsymbol{\omega } dt - \boldsymbol{\omega }\cdot \nabla u dt =\Delta \boldsymbol{\omega } dt \end{aligned}$$

corresponding to a fluid which evolves with velocity *u* and vorticity given by $\boldsymbol{\omega } = curl \ u$. The associated SPDE will have the form

1.3 $$\begin{aligned} dX_t = A_t(X_t)dt+B_t(X_t)dW_t \end{aligned}$$

where the diffusion operator *B* will be constructed such that one can reduce the norm of the system below a suitably chosen value, and *W* is a Wiener process on a probability space $(\Omega , {\mathcal {F}}, {\mathbb {P}})$. For the vorticity equation (1.2) for instance, a possible choice for the diffusion term is $B(\boldsymbol{\omega })=\theta \Vert \boldsymbol{\omega }\Vert ^\alpha \boldsymbol{\omega }$, where $\Vert \boldsymbol{\omega }\Vert$ is a certain Sobolev norm of $\boldsymbol{\omega }$ and $\alpha ,\theta$ are constants that match the nonlinearity in the equation.

The stochastic term stops the blow-up through a “second order” effect. To be more precise, the increase of the norm of the solution of the partial differential equation is counteracted by a term driven by the quadratic variation of the stochastic term. As it is well-known, the stochastic term does not have finite variation paths. When the chain rule is applied for a given function φ of the norm of the solution, an additional term appears as a result of the infinite variation of the stochastic term. This term involves the second derivative of φ. So whilst the nonlinear term appearing in the PDE is multiplied by the first derivative, the quadratic variation term is multiplied by the second derivative. To balance the two, one needs a function that changes signs when differentiated. In the literature on blow-up of (one-dimensional) SDEs the function that has this property is called the *scale function*: more precisely, for a one-dimensional SDE the scale function φ is chosen so that the drift term of the process given by the function of the solution, vanishes (see e.g. [15]). That means that φ will satisfy a second order partial differential equation. Once the drift is eliminated, the remaining stochastic term behaves like a Brownian motion with a time clock depending on its quadratic variation. Explicit conditions can then be given to show when the blow-up will occur.

For an infinite-dimensional SDE (in other words, an SPDE), we cannot apply this procedure directly. The reason for this is that, in general, the drift term depends not only on the norm we are trying to prevent from blowing up, but also on an additional norm of the solution. Depending on the specific case, this can be stronger or weaker than the existence norm. We seek a scale function for the norm of the solution of the SPDE. This scale function, denoted by φ, can no longer be obtained as a solution of a PDE. Instead, we look for a scale function that makes the drift uniformly bounded from above. In the classical case this will mean that the solution of the one dimensional equation might still blow up to -∞. Here the quantity that we want to control is a norm which, by definition remains nonnegative, hence there is no risk of negative blow-up.

Once we remove the constraint of having a null drift, we end up with a large class of stochastic noises that can be used. This is still not enough to stop the blow-up of the norm. Whilst the drift term will have an upper bound of the form *mt* (where *m* is the norm itself and *t* is time), the stochastic term (denoted by) ${\tilde{M}}$ is a local martingale that might still blow up in finite time. As a result, we seek a scale function that will not only bound the drift by *mt*, but it will make it less than $mt-\epsilon \langle \tilde{M}\rangle _t$, where $\langle {\tilde{M}}\rangle$ is the quadratic variation of the local martingale ${\tilde{M}}$ and ϵ is a positive constant. Consequently, the scale function of the norm will now be bounded *pathwise* by $mt + \tilde{M}_t-\epsilon \langle {\tilde{M}}\rangle _t$, as $t\rightarrow \tilde{M}_t-\epsilon \langle {\tilde{M}}\rangle _t$ is bounded on the whole interval [0,∞) by the supremum of a Brownian motion plus a negative constant, which can be controlled ${\mathbb {P}}$ - almost surely (see Proposition 17).

Although not used directly in our analysis, we should also mention that the stochastic term in equation (1.3) is “infinitesimally unbiased” in the sense that it is a local martingale. However, it is not a proper martingale, as we do not know if it is integrable. This poses a major hurdle in the analysis of the solution of the SPDE: all terms can only be controlled in probability *but* not in expectation. We cannot guarantee that the norm process has finite second moment or even that it has finite mean. This difficulty affects all steps in the construction of the global solution of the equation (1.3): the a priori bounds, the tightness results, and the convergence of the solution of the approximating equation.

The results presented below can be used to introduce a (stochastic) control for a dynamical system modelled by the solution of the PDE. We describe just the intuitive idea, and refer to e.g. [4] for more details. The application of the control is done only when the norm of the solution of the PDE exceeds a certain level. The control of the norm is possible as the scale function is chosen not only to make the drift term less than $mt-\epsilon \langle {\tilde{M}} \rangle _t$, but in fact less than $-\epsilon \langle {\tilde{M}} \rangle _t$ for values of the norm larger than a suitable chosen constant *K*. This permits the following stochastic control strategy: we denote by $\tau _0$ the first (deterministic) time when the norm of the solution of the PDE reaches level $\varphi ^{-1}(2K)$, where *K* is the constant defined above and φ is the scale function (e.g. $\varphi (x) = \log x$). If $\tau _0=\infty$, then no control is needed. However, if $\tau _0<\infty$, we apply the stochastic control to the PDE. We keep the control until the norm of the solution, goes down to level $\varphi ^{-1}(K)$. We denote this (stopping) time by $\rho _0$. Once the norm reaches level $\varphi ^{-1}(K)$ we remove the stochastic control and let the equation evolve deterministically until again it reaches level $\varphi ^{-1}(2K)$ (denote the new stopping time by $\tau _1$) at which point we apply the stochastic control again until the solution reaches level $\varphi ^{-1}(K)$ (denote the new stopping time by $\rho _1$) and repeat the procedure. The resulting process is a (strong) Markov process built by piecing together deterministic trajectories and stochastically controlled ones. The general definition of the (stopping) times is as follows

1.4 $$\begin{aligned} \tau _i=\inf \{t\ge \rho _{i-1} | \ \ ||X_t||\ge \varphi ^{-1}(2K) \}, \ \ \ \rho _i=\inf \{t\ge \tau _{i} | \ \ ||X_t||\le \varphi ^{-1}(K) \}.\nonumber \\ \end{aligned}$$

By convention, we choose $\rho _{-1}:=0$. As explained above, the system runs according to the original PDE (1.1) in the intervals $[\rho _{i-1}, \tau _i]$ and according to the SPDE (1.3) on the intervals $[\tau _i, \rho _{i}]$. Note that in all cases below, it takes time for the norm of the solution of the PDE to reach level $\varphi ^{-1}(2K)$ starting from level $\varphi ^{-1}(K)$. More precisely, one can show that there exists a positive constant α which depends only on *K* such that $\tau _i - \rho _{i-1} >\alpha$. This ensures that $\lim _{i\rightarrow \infty }\tau _i=\lim _{i\rightarrow \infty }\rho _i=\infty$. This guarantees that the process *X* is defined on the whole time line [0,∞].

Note that if the stochastic control is introduced at time $\tau _i$, the norm of the process will reach level $\varphi ^{-1}(K)$ in finite time. In other words, $\rho _i-\tau _i<\infty$, ${\mathbb {P}}-a.s.$. As stated above, observe that on the interval $[\tau _i,\rho _i]$ the upper bound for the process $t\rightarrow \varphi (\Vert X_t\Vert )$ is the process $t\rightarrow 2K+{\tilde{M}}_t-\epsilon \langle \tilde{M}\rangle _t$. This process is, in turn, a time-changed Brownian motion with negative drift which will eventually hit level φ(K) as the time change tends to infinity. This argument corresponds to Case I and Case II described in Section 2 below.

In the third case described in Section 2 below, it may be possible for $\rho _i$ to be ∞. The fix in this case is to choose a higher level *K* which will ensure that the drift term of the scale function applied to the norm $t\rightarrow \varphi (\Vert X_t\Vert )$ has an upper bound of the form $t\rightarrow 2K-\delta (t-\tau _i) + {\tilde{M}}_t-\epsilon \langle {\tilde{M}}\rangle _t$ which will in turn ensure that $t\rightarrow \varphi (\Vert X_t\Vert )$ eventually reaches level *K*.

We emphasize that the blow-up control obtained through the stochastic term does not act in a way similar to a forcing term. More precisely, it is the quadratic variation of the stochastic term that stops the blow-up. In this sense this can be viewed as a second order effect as the stochastic term does not directly influence the blow-up. The system becomes infinitesimally unbiased due to the quadratic variation. To understand this, let us look at the following linear one-dimensional ODE (we write it in differential term as we will add a stochastic term to it):

$$\begin{aligned} df_t=af_t dt, \ \ \ \ f_0,a>0. \end{aligned}$$

The solution of this ODE does not blow-up, but it does increase exponentially fast in time: $f_t=f_0\exp (at)$. Let us contrast this with the solution of the same equation but now stochastically perturbed:

$$\begin{aligned} df_t=af_t dt+bf_t dW_t, \ \ \ \ f_0,a>0, \end{aligned}$$

where we get a one dimensional geometric Brownian motion, with the explicit solution

$$\begin{aligned} f_t=f_0\exp \left( at-{\frac{b^2}{2}}\langle W\rangle _t+bW_t\right) =f_0\exp \left( \left( a-{\frac{b^2}{2}}\right) t+bW_t\right) \end{aligned}$$

where $\langle W \rangle =t$ is the quadratic variation of the Brownian motion *W*. We deduce immediately that, as soon as the diffusion coefficient is sufficiently high (in other words as soon as we inject sufficient noise into the system), more precisely, if we choose $b^2>2a$ then the solution converges to 0 asymptotically. The effect of the stochasticity is severe: from exponential increase we end up with exponential decay to 0. However we should note that the expected value of the geometric Brownian motion still increases exponentially (in fact it satisfies the deterministic PDE - the perturbation is unbiased) even though, with probability 1, every path decays to 0. It is this effect of the stochasticity that we exploit to control the blow-up of the (S)PDEs.

We should further remark that the control we need is more severe than the exponential increase described above. More precisely we need to control a (possible) blow-up in finite time. For this reason, we cannot expect to be able to use a linear/multiplicative noise. We will need instead a super-linear noise. This will have several secondary effects:

- The resulting stochastic term will **not** be a martingale but just a local martingale. We cannot even prove that it has finite expectation. All uniform controls, tightness and convergence results will have to be adapted to cope with this difficulty. The standard tightness criteria need to be modified to account for these difficulties.
- The control is not unique: as long as the superlinearity is sufficiently high, we obtain the desired result.

Several attempts have been made to show improved properties of SPDEs driven by various type of noise, compared to their deterministic counterparts (see e.g. [6, 13, 10, 18]). However, due to its intrinsic structure, transport noise seems to leave the turbulent behaviour of the fluid untouched. It becomes therefore interesting to look at different noise structures which could better explain the uncertainty in a turbulent dynamical system. We make a step forward by proposing a noise structure which can at least improve the analytical properties of the system. The physical implications of this type of noise are perhaps less clear. Our analysis requires a fine balance between the conditions imposed on the nonlinear operator, the initial conditions, and the choice of the stochastic term. Only in this way we succeed to show a priori bounds on the solution of the SPDE, the tightness of the approximating sequence and its convergence. The results we obtain can cover a wide class of models from stochastic fluid dynamics: compressible **and** incompressible, viscous **and** inviscid.

Formally, we work with four separable Hilbert spaces ${\mathscr {D}}, {\mathscr {F}}_1, {\mathscr {F}}_0, {\mathscr {G}}$ which are compactly embedded in each other

1.5 $$\begin{aligned} {\mathscr {D}} \hookrightarrow {\mathscr {F}}_1 \hookrightarrow {\mathscr {F}}_0 \hookrightarrow {\mathscr {G}}. \end{aligned}$$

Typically, the main space of existence of the corresponding solution is ${\mathscr {F}}_0$, endowed with the uniform norm in time, when a higher order integral of the ${\mathscr {F}}_1$ norm also exists (this setting is characteristic to models which contain a viscous term). The space ${\mathscr {D}}$ is a higher order space in which we can control the norm of an approximating sequence of solutions uniformly, while the space ${\mathscr {G}}$ is the space in which the approximating sequence of solutions converges to the solution of the original equation. We identify three distinct cases, depending formally on the space where the initial condition lives and the form of the noise term, see (2.13)-(2.15) in Section 2. However, this structure is actually dictated by the form of the nonlinear drift and the required regularity of the solution. The second case (see (2.14)) corresponds to a stochastic closure of a compressible inviscid model and it is, from this perspective, the most difficult. This is natural, as this is the case where the noise truly improves the properties of the solution - the associated deterministic system cannot be closed without adding artificial viscosity (or a different type of transformation) and to the best of our knowledge, no global existence result exists for such a system. So by adding the right type of stochasticity, we overcome this long-standing issue in the theory of compressible inviscid fluid dynamics models.

It is, a priori, not clear how “physical” is the noise we propose. We know that there is usually a delicate balance between the energy which is created within a multi-scale nonlinear system and the energy which is dissipated through the diffusion term. The blow-up occurs when this balance is broken, and our noise term is designated in a special way to provide an exact balance between absorbed and dissipated energy. That is, the stochastic term extracts “the right amount of energy” for the solution to be stabilised. Note that the model we present is nonlocal, the noise depending on the whole domain.

The interest in proving existence of global strong solutions has been continuously growing over the last years. In some sense, our result is closest in spirit to [26] where the authors prove existence of global solutions in a finite-dimensional setting: we extend this result to the infinite-dimensional case, and adapt it to a much more abstract functional setting. In [21] it has been proven that an explicit Stratonovich-type noise can prevent blow-up: this holds on ${\mathbb {R}}^d$ with d≥2, and under specific super-linear growth assumptions on the drift. In [2] the theory from [21] is extended such that the existence of a global-in-time strong solution for stochastic Euler-type equations in 2D and 3D can be shown. In [25] the authors prove that for Camassa-Holm-type equations, a strong enough noise can prevent the blow-up with probability 1, with a specific dependence on the initial conditions. In [1] it has been shown that uniqueness and almost sure existence of solutions can be proven for a one dimensional transport equation under random perturbations on the real line.

Alternative approaches for showing existence of a global solution involve so-called regularisation/α-regularisation methods: see e.g. [3, 22, 9]. However, we are not going in this direction, as our aim is to find a noise term which makes the solution global, not to prove scaling limits or to prove existence of a global solution for a deterministic equation.

Although our results exhibit certain similarities with previous works, they were obtained independently as part of a broader approach. Our goal was not merely to suppress the blow-up through a suitable stochastic component-an improvement in itself-but to leverage the stochastic term to achieve even more: ensuring the existence of a solution in the stochastic setting where no solution is known to exist in the original deterministic case (see e.g. the inviscid rotating shallow water system). This provides further evidence that stochasticity, when carefully defined, can indeed enhance the analytical properties of equations, and highlights its particular relevance for compressible models. We identify the appropriate analytical form of the stochastic term to achieve such improvements, thereby contributing to a more well-founded model structure. Our approach was motivated by our own earlier work in [7, 17], and [16], which has been carried out before the publication of [2].

In [17] we have shown existence of a global *weak* (in PDE sense) solution, while the existence of a *strong* solution could only be proven locally. In [7], on the other hand, we have proven that a global strong solution can exist with positive probability. That is, if ${\mathcal {T}}$ is the corresponding maximal time of existence, we have ${\mathbb {P}}({\mathcal {T}} = \infty ) > 0$. However, in this paper we show that in such a case we have ${\mathbb {P}}({\mathcal {T}} = \infty ) = 1$.

In Section 2 we start by introducing the setting and the main assumptions. In Section 3 we present the main results, which are proven using the uniform control shown in Section 3.1 and the tightness result presented in Section 3.2. In Section 4 we list several examples from fluid dynamics to which our theory applies.

## Setting and assumptions

Let $(\Omega , {\mathcal {F}}, ({\mathcal {F}}_t)_t, {\mathbb {P}})$ be a fixed stochastic basis. Let ${\mathscr {D}}, {\mathscr {F}}_1, {\mathscr {F}}_0, {\mathscr {G}}$ be separable Hilbert spaces such that

2.1 $$\begin{aligned} {\mathscr {D}} \hookrightarrow {\mathscr {F}}_1 \hookrightarrow {\mathscr {F}}_0 \hookrightarrow {\mathscr {G}} \end{aligned}$$

are compact embeddings with

2.2 $$\begin{aligned} {}_{{\mathscr {D}}}\langle a, b\rangle _{{\mathscr {G}}} = \langle a, b \rangle _{{\mathscr {F}}_i} \quad \hbox {for all} \quad a\in {\mathscr {D}}, b\in {\mathscr {F}}_i \end{aligned}$$

and there is C>0 and m∈(0,1) such that

$$\begin{aligned} \Vert f\Vert _{{\mathscr {F}}_0} \le C\Vert f\Vert _{{\mathscr {F}}_1}^{m}\Vert f\Vert _{{\mathscr {G}}}^{1-m} \quad \quad \text{ for } \text{ all } \quad f\in {\mathscr {F}}_1. \end{aligned}$$

Let ${\mathscr {U}}$ be another separable Hilbert space. We introduce the following operators

2.3 $$\begin{aligned}  &   A:[0,T] \times {\mathscr {F}}_{i} \times \Omega \rightarrow {\mathscr {G}} \end{aligned}$$

2.4 $$\begin{aligned}  &   B: [0,T] \times {\mathscr {F}}_{i} \times \Omega \rightarrow L_2({\mathscr {U}},{\mathscr {F}}_i) \end{aligned}$$

which are progressively measurable with respect to ${\mathcal {B}}([0,t])\times {\mathcal {B}}({\mathscr {F}}_i)\times {\mathcal {F}}_t$ for any t∈[0,T] and $T\in [0,\infty )$ fixed. Above we have either i=0 or i=1, depending on the structure of the drift, and $L_2({\mathscr {U}},{\mathscr {F}}_i)$ is the space of Hilbert-Schmidt operators from ${\mathscr {U}}$ to ${\mathscr {F}}_i$. In what follows, we will resume to the particular case when ${\mathscr {U}}$ is one-dimensional, in other words, ${\mathscr {U}} \cong {\mathbb {R}}$. That is because considering a real-valued driving Wiener process ensures the control we require for establishing global existence. In this case, one has the canonical identification $L_2({\mathscr {U}},{\mathscr {F}}_i) \cong {\mathscr {F}}_i$ such that the operator *B* may be regarded as ${\mathscr {F}}_i$-valued. To keep the notation consistent with the general SPDE framework, and to emphasize that no essential obstruction arises in the case of higher or infinite-dimensional Wiener processes, we will continue to write $B \in L_2({\mathscr {U}},{\mathscr {F}}_i)$, although this additional generality is not required for the arguments below.

Consider the following SPDE on ${\mathscr {G}}$:

2.5 $$\begin{aligned} dX_t = A_t(X_t)dt + B_t(X_t)dW_t \end{aligned}$$

where *W* is a one-dimensional real-valued Wiener process and $X_0$ is a ${\mathcal {F}}_0$-measurable ${\mathscr {F}}_i$-valued random variable. We assume that this equation admits a unique maximal strong solution $(X, {\mathcal {T}})$ in ${\mathscr {F}}_i$ in the sense described in Section 3. We define

2.6 $$\begin{aligned} B_t(X_t):= \theta \displaystyle \Vert X_t\Vert _{{\mathscr {F}}_{i}}^{\alpha _{i}}X_t, \end{aligned}$$

where $\alpha _{i}, \theta \in {\mathbb {R}}_{+}$, and *i* will be either 0 or 1 depending the conditions imposed on the operator *A*. In other words, equation (2.5) takes the following form:

2.7 $$\begin{aligned} dX_t = A_t(X_t)dt + \theta \displaystyle \Vert X_t\Vert _{{\mathscr {F}}_{i}}^{\alpha _i}X_tdW_t. \end{aligned}$$

Let $(\rho _i)_i$ be an orthogonal basis in ${\mathscr {D}}$ and denote by ${\mathscr {D}}^d$ the span generated by $\rho _1, \rho _2, \ldots , \rho _d$. So

2.8 $$\begin{aligned} {\mathscr {D}}^d = span\{\rho _1, \rho _2, \ldots , \rho _d\}. \end{aligned}$$

We denote by $T_d$ the projection operator $T_d: {\mathscr {G}} \rightarrow {\mathscr {D}}^d$.^1^ Then denote by $A^d$ and $B^d$ respectively the projection of the operators *A* and *B*. That is

2.9 $$\begin{aligned} \begin{aligned}&A^d(X^d) := T_d(A(X^d)) \\&B^d(X^d) := T_d(B(X^d)) \end{aligned} \end{aligned}$$

with $A^d, B^d:{\mathscr {D}}^d \rightarrow {\mathscr {D}}^d$. We assume that the embedding ${\mathscr {D}} \hookrightarrow {\mathscr {F}}_i$ is compact, continuous, and dense, the embedding ${\mathscr {F}}_i \hookrightarrow {\mathscr {G}}$ is continuous and dense, and $span\{(\rho _i)_i\}$ is dense in ${\mathscr {G}}$, while *i* can be either 0 or 1 depending on the structure of the drift (see the three cases below). So ${\mathscr {D}}$ is a dense subspace of ${\mathscr {G}}$. A global solution for equation (2.5) will be constructed using a *d*-dimensional projected equation:

2.10 $$\begin{aligned} dX_t^d = A_t^d(X_t^d)dt + \theta \displaystyle \Vert X_t^d\Vert _{{\mathscr {F}}_{i}}^{\alpha _{i}}X_t^d dW_t^d \quad \hbox {on} \quad [0,T] \end{aligned}$$

where $X_t^d$ is the classical *d*-dimensional (Galerkin) projection of $X_t$^2^ and $W^d$ is a one-dimensional Brownian motion. Equation (2.10) admits a global solution: this is a direct consequence of Theorem 2.4 and Theorem 2.5 in [26]. An alternative argument is based on the fact that since equation (2.10) has locally Lipschitz coefficients, it therefore has a unique maximal solution. In other words, (2.10) has a unique solution $X^d$ defined up to a (maximal) stopping time $\tau _{max}$. Should $\tau _{max}<\infty$ it would follow that the solution blows up, but this is not possible, as under the conditions imposed below, the corresponding a priori estimates are *d*-independent (see e.g. Section 3.1), and then

$$\begin{aligned} \displaystyle \lim _{\ell \rightarrow \infty } {\mathbb {P}}\left( \tau _{\ell } < t\right) = 0 \end{aligned}$$

for any local solution which exists up to the corresponding stopping time $\tau _{\ell }$ and such that $\displaystyle \lim _{\ell \rightarrow \infty }\tau _{\ell } = \tau _{max}$. So necessarily $\tau _{max} = + \infty$. This is also the basis of the arguments presented in [26]. We introduce next the various types of solutions used in this paper:

### Definition 1

(Definition of solutions) Let ${\mathcal {S}} = \left( \Omega , {\mathcal {F}}, ({\mathcal {F}}_t)_t, {\mathbb {P}}, (W_t)_t\right)$ be a fixed stochastic basis.

- A pathwise local analytically strong solution to (2.5) is given by a pair (X,τ) where $\tau :\Omega \rightarrow [0,\infty ]$ is a strictly positive bounded stopping time, $X=(X_t)_t$ is an ${\mathscr {F}}_i$-valued process such that $X_{\cdot \wedge \tau }$ is $({\mathcal {F}}_{t})_t$-adapted for any t≥0 and (2.5) is satisfied locally i.e.
  $$\begin{aligned} X_{t\wedge \tau }=X_{0}+\int _{0}^{_{t\wedge \tau }}A_s(X_{s}) ds+\int _{0}^{_{t\wedge \tau }}B_s(X_{s}) dW_{s} \end{aligned}$$
  holds ${\mathbb {P}}$-almost surely as an identity in ${\mathcal {G}}$.
- If $\tau =\infty$ then the solution is called global.
- A pathwise maximal solution to (2.5) is given by a pair $(X, {\mathcal {T}})$ where ${\mathcal {T}}: \Omega \rightarrow [0, \infty ]$ is a non-negative stopping time and $X=(X_t)_t$ is an ${\mathscr {F}}_i$-valued process for which there exists an increasing sequence of stopping times $(\tau ^n)_n$ with the following properties:
  i. $${\mathcal {T}}=\lim _{n\rightarrow \infty }\tau ^{n}$$ and $${\mathbb {P}}( {\mathcal {T}}>0)=1$$
  ii. $(X,\tau ^{n})$ is a pathwise local solution to (2.5) for every $n\in {\mathbb {N}}$
  iii. if ${\mathcal {T}}<\infty$ then
    $$\begin{aligned} \displaystyle \limsup _{t \rightarrow {\mathcal {T}}} \Vert X_{t}\Vert _{{\mathscr {F}}_0}=\infty . \end{aligned}$$
- A global analytically weak solution to (2.5) is given by an $({\mathcal {F}}_t)_t$-adapted process $X=(X_t)_t$ such that the following identity holds for any test function $\varphi \in {\mathscr {D}}$:
  $$\begin{aligned} X_t(\varphi )= &   \langle X_t, \varphi \rangle \\= &   \langle X_0,\varphi \rangle + \displaystyle \int _0^t {}_{{\mathscr {G}}}\langle A_s(X_s),\varphi \rangle _{{\mathscr {D}}} ds + \displaystyle \int _0^t {}_{L_2({\mathscr {U}},{\mathscr {F}}_i)}\langle B_s(X_s),\varphi \rangle _{{\mathscr {D}}} dW_s. \end{aligned}$$
- The process $X=(X_t)_t$ is a martingale solution to (2.5) if there exists a filtered probability space $\left( \tilde{\Omega }, \tilde{{\mathcal {F}}}, (\tilde{{\mathcal {F}}}_t)_t, \tilde{{\mathbb {P}}}\right)$ with a standard Wiener process $\tilde{W}=(\tilde{W}_t)_t$ and an initial condition $\tilde{X}_0$ defined on it, such that $\tilde{X}_0$ and $X_0$ have the same distribution and $X_t$ is an $(\tilde{{\mathcal {F}}}_t)_t$-adapted solution on $\tilde{\Omega }$, to the following equation
  2.11 $$\begin{aligned} dX_t = A_t(X_t)dt + B_t(X_t)d\tilde{W}_t \end{aligned}$$
  with initial condition $\tilde{X}_0$.

### Remark 2

Note that an analytically weak solution *X* becomes analytically strong provided

2.12 $$\begin{aligned} \begin{aligned}&{\mathbb {P}}\left( \displaystyle \int _0^T \Vert A_s(X_s)\Vert _{{\mathscr {F}}_i}ds<\infty \right) = 1 \\&{\mathbb {P}}\left( \displaystyle \int _0^T \Vert B_s(X_s)\Vert _{L_2({\mathscr {U}},{{\mathscr {F}}_i)}}^2ds<\infty \right) = 1. \end{aligned} \end{aligned}$$

Our main result says, informally, that

*Under suitable assumptions and given the embeddings* (2.1)*, equation* (2.7) *admits a global strong solution in* ${\mathscr {F}}_0$*,* ${\mathscr {F}}_1$*, and* ${\mathscr {D}}$*, respectively*.

The precise statement of this result is given in Theorem 7 below. We distinguish three main cases:

**Case I:** The initial condition $X_0\in {\mathscr {F}}_0$, θ>0, assumption (A1) below is satisfied, and

2.13 $$\begin{aligned} B_t(X_t) = \theta \Vert X_t\Vert _{{\mathscr {F}}_0}^{\alpha _0}X_t. \end{aligned}$$

**Case II:** The initial condition $X_0 \in {\mathscr {D}}$, θ>0, assumption (A2) below is satisfied, and

2.14 $$\begin{aligned} B_t(X_t) = \theta \Vert X_t\Vert _{{\mathscr {F}}_1}^{\alpha _1}X_t. \end{aligned}$$

**Case III:** The initial condition $X_0 \in {\mathscr {F}}_1$, θ>0, assumption (A3) below is satisfied, and

2.15 $$\begin{aligned} B_t(X_t) = \theta \Vert X_t\Vert _{{\mathscr {F}}_0}^{\alpha _0}X_t. \end{aligned}$$

### Remark 3

Typical models which can be covered by Case I are compressible and incompressible viscous models (e.g. viscous rotating shallow water models), while typical models covered by Case II and Case III are compressible inviscid (e.g. inviscid rotating shallow water model) and, respectively, incompressible models (e.g. 3D Euler equation). For explicit details regarding these connections and further examples, see Section 4.

### Remark 4

We emphasize that the role of the space ${\mathscr {F}}_1$ in Case III is, in a certain sense, different from the role of the same space in the first two cases and it should be read in the following key: in Case III ${\mathscr {F}}_1$ **is** the main space of existence of the solution, and the noise depends on the norm of the solution in a larger space, that is the ${\mathscr {F}}_0$ norm. We could choose the space of existence to be ${\mathscr {F}}_0$ as in the previous two cases, but then we would need to introduce a new larger space between ${\mathscr {F}}_0$ and ${\mathscr {G}}$, say ${\mathscr {F}}^{\star }$ which would be used to construct the stochastic term. The analysis is identical in any of these two situations, so we decided not to introduce an extra space, and to proceed with the analysis in the quadruple of spaces ${\mathscr {D}}\hookrightarrow {\mathscr {F}}_1 \hookrightarrow {\mathscr {F}}_0 \hookrightarrow {\mathscr {G}}$. Note that in order to stop the blow-up in a generic functional space ${\mathscr {F}}^{\star }$ we need to make sure that the evolution of the corresponding solution can first be controlled in a smaller/higher order space.

### Remark 5

It must be emphasized that for the compressible inviscid model we cannot replace the control in ${\mathscr {F}}_0$ with control in ${\mathscr {D}}$, as for showing control in ${\mathscr {D}}$ we need control in ${\mathscr {F}}_0$. That is, overall we need all four spaces ${\mathscr {D}}, {\mathscr {F}}_1, {\mathscr {F}}_0$ and ${\mathscr {G}}$, and we use both Proposition 11 and Proposition 13 to ensure the required uniform control.

In order to obtain global existence, the following assumptions will need to be satisfied by the *d*-dimensional approximation $(X^d)_d$:

- **Assumption (A1):** The initial condition is in ${\mathscr {F}}_0$ and for $a\in {\mathscr {D}}^d$ it holds that
  2.16 $$\begin{aligned} \langle a, A^d(a)\rangle _{{\mathscr {F}}_0} \le C_1 \Vert a\Vert _{{\mathscr {F}}_0}^{\gamma _1} -C_2\Vert a\Vert _{{\mathscr {F}}_1}^{2} \end{aligned}$$
  where $C_1, \gamma _1$ are positive constants and $C_2 \in {\mathbb {R}}$. For $a\in {\mathscr {F}}_1$
  2.17 $$\begin{aligned} \Vert a\Vert _{{\mathscr {F}}_0} \le C_0\Vert a\Vert _{{\mathscr {F}}_1}^{\alpha }\Vert a\Vert _{{\mathscr {G}}}^{\beta } \end{aligned}$$
  and
  2.18 $$\begin{aligned} \Vert A^d(a)\Vert _{{\mathscr {G}}} \le C_1\Vert a\Vert _{{\mathscr {F}}_0}^{\gamma ^1}+ C_2\Vert a\Vert _{{\mathscr {F}}_1}^{\gamma ^2}+C_3, \end{aligned}$$
  where $\alpha , \beta , \gamma ^i, C_i, i\in \{0, 1,2,3\}$ are positive constants. Moreover, for any $a,b \in {\mathscr {F}}_0$
  2.19 $$\begin{aligned} \Vert A^d(a) - A^d(b)\Vert _{{\mathscr {G}}} \le C(1+\Vert a\Vert _{{\mathscr {F}}_1}+ \Vert b\Vert _{{\mathscr {F}}_1})\Vert a - b\Vert _{{\mathscr {F}}_0} \end{aligned}$$
  with *C* a positive constant.
- **Assumption (A2):** The initial condition is in ${\mathscr {D}}$, (2.16), (2.19), and the following conditions hold:
  2.20 $$\begin{aligned}  &   \langle a, A^d(a)\rangle _{{\mathscr {D}}} \le C_1 \Vert a\Vert _{{\mathscr {F}}_1}^{\gamma _1}\Vert a\Vert _{{\mathscr {D}}}^{\gamma _2} \end{aligned}$$
  2.21 $$\begin{aligned}  &   \Vert a\Vert _{{\mathscr {F}}_1} \le C\Vert a\Vert _{{\mathscr {D}}}^{\alpha }\Vert a\Vert _{{\mathscr {G}}}^{\beta } \end{aligned}$$
  where $C_1, C, \gamma _1, \gamma _2, \alpha , \beta$ are positive constants. Condition (2.18) holds with $C_2=0$. We also assume that the approximating equation (2.10) admits a solution in ${\mathscr {F}}_0$ which does not hit zero or, if it hits zero, it remains zero.
- **Assumption (A3):** The initial condition is in ${\mathscr {F}}_1$ and the following conditions hold:
  2.22 $$\begin{aligned}  &   \langle a, A^d(a)\rangle _{{\mathscr {F}}_1} \le C_1 \Vert a\Vert _{{\mathscr {F}}_0}^{\gamma _{13}}\Vert a\Vert _{{\mathscr {F}}_1}^2 \end{aligned}$$
  2.23 $$\begin{aligned}  &   \Vert a\Vert _{{\mathscr {F}}_0} \le C\Vert a\Vert _{{\mathscr {F}}_1}^{\alpha }\Vert a\Vert _{{\mathscr {G}}}^{\beta } \end{aligned}$$
  where $C_1, C, \gamma _{13}, \alpha , \beta$ are positive constants. Condition (2.18) holds with $C_1=0$. Moreover, for any $a,b \in {\mathscr {F}}_0$
  2.24 $$\begin{aligned} \Vert A^d(a) - A^d(b)\Vert _{{\mathscr {G}}} \le C(1+\Vert a\Vert _{{\mathscr {F}}_1}+ \Vert b\Vert _{{\mathscr {F}}_1})\Vert a - b\Vert _{{\mathscr {F}}_1} \end{aligned}$$
  with *C* a positive constant.

### Remark 6

- In practice, condition (2.16) can be obtained directly from a condition of the form
  2.25 $$\begin{aligned} {}_{{\mathscr {D}}}\langle a, A^d(a)\rangle _{{\mathscr {G}}} \le C_1 \Vert a\Vert _{{\mathscr {F}}_0}^{\gamma _1} + C_2\Vert a\Vert _{{\mathscr {F}}_1}^{\gamma _2} \end{aligned}$$
  which is natural for a nonlinear drift which contains a higher order term (and frequently with $\gamma _2=2$). The equivalence of the scalar products $\langle \cdot ,\cdot \rangle _{{\mathscr {F}}_0}$ and ${}_{\mathscr {D}}\langle \cdot , \cdot \rangle _{{\mathscr {G}}}$ is due to condition (2.2).
- We highlight that one does not necessarily need an extra space in case II at the expense of using a smaller space in order to have convergence of the stochastic integral, and in that case the initial condition needs to be in ${\mathscr {D}}$ which is more restrictive.
- It is noteworthy that conditions such as (2.16), (2.202.21), and (2.222.23) are natural for geophysical fluid dynamics models. Condition (2.222.23), for instance, can be verified for an incompressible model: the fact that the ${\mathscr {F}}_1$-norm appears on both sides is possible due to the incompressibility condition. Unlike that, condition (2.202.21) can be verified for a compressible inviscid model, but the fact that the ${\mathscr {D}}$-norm appears on both sides is possible due to the finite-dimensionality of the space ${\mathscr {D}}^d$ which is dense in ${\mathscr {D}}$ and which is the space of existence for the finite-dimensional approximating system. Condition (2.16) is, in some sense, typical for compressible viscous models with ${\mathscr {F}}_0$-valued solutions (see particular cases in e.g. [17] and [7]). We call *“space of existence”"* the space where the uniform norm is controlled. This is just a shortened terminology that we use here, as one can notice that the full functional spaces where the respective solutions live is $L^{\infty }\left( (0,T), {\mathscr {I}}\right)$ where ${\mathscr {I}}$ is equal to ${\mathscr {F}}_0$, ${\mathscr {F}}_1$ or ${\mathscr {D}}$ endowed, respectively, with the following norms:
  $$\begin{aligned} \begin{aligned}&{{\textbf {Case I}}} \quad \quad \quad ||| X |||_{{\mathscr {F}}_{0},{\mathscr {F}}_{1},T}^{2}:=\sup _{t\in \left[ 0,T\right] }\Vert X_{t}\Vert _{{\mathscr {F}}_{0}}^2+\int _{0}^{T}\Vert X_{s}\Vert _{{\mathscr {F}}_{1}}^{2}ds\\&{{\textbf {Case II}}} \quad \quad \quad |||X|||_{{\mathscr {D}},T}:=\sup _{t\in \left[ 0,T\right] }\Vert X_{t}\Vert _{{\mathscr {D}}}\\&{{\textbf {Case III}}} \quad \quad \quad |||X|||_{{\mathscr {F}}_{1},T}:=\sup _{t\in \left[ 0,T\right] }\Vert X_{t}\Vert _{{\mathscr {F}}_{1}}. \end{aligned} \end{aligned}$$
- In condition (2.16) we have $C_2 > 0$ for compressible viscous models such as viscous rotating shallow water (Case I) and $C_2 < 0$ for compressible inviscid models such as standard (inviscid) rotating shallow water (Case II). This is because in the second case there is no dissipation in the original deterministic drift. Instead, we "create" dissipation from the stochastic term, to counter-balance the effect of the nonlinear term. In order to further ensure global existence, the intrinsic properties of the stochastic term are instrumental.
- Under similar conditions, in a sequel to this work we will show that the (time-homogeneous version of the) process *X* which solves the equation has an invariant measure on ${\mathscr {G}}$. To do this we will show that *X* is a Markov process and that its corresponding semigroup $\left\{ P_{t},t\ge 0\right\}$ is Feller. Moreover, for an arbitrary μ on ${\mathscr {G}}$ we define the set of measures
  $$\begin{aligned} \mu _{t}(A)=\frac{1}{t}\int _{0}^{t}\int _{{\mathscr {G}}}P(s,x,A)ds\mu (dx),~~t\ge 0 \end{aligned}$$
  for all $A\in {\mathscr {B}}({\mathscr {G}})$. Then we show that $\left\{ \mu _{t}\right\} _{t\ge 0}$ is tight and that any weak limit of a subsequence $\left\{ \mu _{t_{n}}\right\} _{t_{n}\ge 0}$ such that $\left\{ t_{n}\right\} _{n=1}^{\infty }\subset {\mathbb {R}}_{+},t_{n}\rightarrow \infty$ is an invariant measure for *X*. The key result used to prove tightness is the control given in Proposition 11.
- Note that for showing uniqueness we would need a locally Lipschitz condition such as
  2.26 $$\begin{aligned} \Vert A^d(a) - A^d(b)\Vert _{{\mathscr {G}}} \le C(\Vert a\Vert _{{\mathscr {F}}_1}, \Vert b\Vert _{{\mathscr {F}}_1})\Vert a - b\Vert _{{\mathscr {G}}} \end{aligned}$$
  where *C* is continuous as a function of *a* and *b* and it is independent from the dimension *d*. As a consequence, the operator *A* is locally Lipschitz and therefore we have uniqueness of the solution in the original equation. This implies uniqueness of the maximal solution (which we have already assumed) and then one can deduce the uniqueness of the newly-constructed global solution: we know that any maximal solution $\hat{X}$ is unique up to a maximal stopping time ${\mathcal {T}}$. That is $\hat{X} = X$ on $[0,{\mathcal {T}})$. However, we show that *X* itself is a **global** solution. As a consequence, we need to have ${\mathbb {P}}\left( {\mathcal {T}} = \infty \right) =1$. Note also that condition (2.26) is a generalised version of (2.19) and (2.24), so assumptions (A1)-(A3) imply directly uniqueness for the maximal solution.

## Main results

Our main theorem is given below:

### Theorem 7

Let the assumptions (*A*1), (*A*2) and (*A*3) be simultaneously satisfied. Then the stochastic partial differential equation (2.7) admits a global strong solution *X* with paths that belong to the space $C\left( [0,\infty ),{\mathscr {G}}\right) ,$ ${\mathbb {P}}$-almost surely, such that for any T>0 ,

3.1 $$\begin{aligned} {\mathbb {P}}\left( \sup _{t\in \left[ 0,T\right] }\left| \left| X_{t}\right| \right| _{{\mathscr {G}}}<\infty \right) =1 . \end{aligned}$$

### Remark 8

As we shall see, in each of the three cases, one can prove stronger bounds for the process *X* that hold for any T>0. They are as follows:

3.2 $$\begin{aligned}  &   \mathbf {Case \ I: \quad }{\mathbb {P}}\left( \sup _{t\in \left[ 0,T\right] }\left| \left| X_{t}\right| \right| _{{\mathscr {F}}_{0}}+\int _{0}^{T}\left| \left| X_{s}\right| \right| _{{\mathscr {F}} _{1}}^{2}ds<\infty \right) =1. \end{aligned}$$

3.3 $$\begin{aligned}  &   \mathbf {Case \ II: \quad }{\mathbb {P}}\left( \sup _{t\in \left[ 0,T\right] }\left| \left| X_{t}\right| \right| _{{\mathscr {D}}}<\infty \right) =1. \end{aligned}$$

3.4 $$\begin{aligned}  &   \mathbf {Case \ III: \quad }{\mathbb {P}}\left( \sup _{t\in \left[ 0,T\right] }\left| \left| X_{t}\right| \right| _{{\mathscr {F}}_{1}}<\infty \right) =1. \end{aligned}$$

Following from the above controls one can deduce, in each of the three cases, that

3.5 $$\begin{aligned} {\mathbb {P}}\left( \int _{0}^{T}\left| \left| A_{s}(X_{s})\right| \right| _{{\mathscr {G}}}ds+\int _{0}^{T}\left| \left| B_{s}(X_{s})\right| \right| _{{\mathscr {G}}}^{2}ds<\infty \right) =1. \end{aligned}$$

In other words that the process X is a local semimartingale (the martingale part of X is a local martingale and not a genuine martingale). The bound (3.5) also confirms the continuity of the martingale *X* in ${\mathscr {G}}$ and that the solution of (2.7) is indeed strong.

### Remark 9

The common denominator of the above conditions is that

$$\begin{aligned} {\mathbb {P}}\left( \sup _{t\in \left[ 0,T\right] }\left| \left| X_{t}\right| \right| _{{\mathscr {F}}_{0}}\right) =1 \end{aligned}$$

in other words *X* has paths in $L^{\infty }\left( [0,T],{\mathscr {F}} _{0}\right) ,$ ${\mathbb {P}}$-almost surely, for any T>0.

The proof of Theorem 7 will be presented in three parts. More precisely:

### Proof of Theorem 7

In order to prove Theorem 7 we need the uniform control and tightness for the approximating sequence. These are proven below in Section 3.1 and Section 3.2, respectively. By Proposition 14, $$(X^{d})_{d}$$ is tight in $$ {\mathscr {G}}$$, and therefore it has a weakly (in distribution) convergent subsequence which we re-index by $$(X^{d})_{d}$$. By the Skorokhod representation theorem, there exists a probability space $$\left( \tilde{ \Omega },\tilde{{\mathcal {F}}},\tilde{{\mathbb {P}}}\right) $$ on which the processes $$\tilde{X}^{d}$$ are defined and such that they have the same distribution as $$X^{d}$$ and $$\tilde{X}^{d}$$ converges to a process $$\tilde{X} $$, $$\mathbb {\tilde{P}}$$ - almost surely. The sequence $$(\tilde{X} _{t}^{d})_{d}$$ inherits all the distributional properties from the sequence $$ (X^{d})_{d}.$$ In particular the bounds in Propositions (11) and (13) are satisfied and therefore $$(\tilde{X}_{t}^{d})_{d}$$ satisfies the controls (3.2), (3.3), (3.4), (3.5). By Fatou’s lemma also the limiting process $$\tilde{X}$$ satisfies the same controls. Moreover on $$\left( \tilde{\Omega },\tilde{{\mathcal {F}}},\tilde{{\mathbb {P}}}\right) $$ there exists a sequence of Brownian motions $$(\tilde{W}_{t}^{d})_{d}$$ such that

3.6 $$\begin{aligned} \tilde{X}_{t}^{d}=\tilde{X}_{0}^{d}+\displaystyle \int _{0}^{t}\tilde{A} _{s}^{d}(\tilde{X}_{s}^{d})ds+\displaystyle \int _{0}^{t}\tilde{B}_{s}^{d}( \tilde{X}_{s}^{d})d\tilde{W}_{s}^{d} \end{aligned}$$

more precisely

3.7 $$\begin{aligned} \tilde{X}_{t}^{d}=\tilde{X}_{0}^{d}+\displaystyle \int _{0}^{t}\tilde{A} _{s}^{d}(\tilde{X}_{s}^{d})ds+\displaystyle \int _{0}^{t}\theta \displaystyle \Vert \tilde{X}_{s}^{d}\Vert _{{\mathscr {F}}_{i}}^{\alpha _{i}}\tilde{X} _{s}^{d}d\tilde{W}_{s}^{d} \end{aligned}$$

on [0, *T*], with $i\in \{0,1\}$, where $\tilde{A}^d, \tilde{B}^d$ are the operators defined on $[0,T] \times {\mathscr {F}}_i \times \tilde{\Omega }$ with the same properties as the operators $A^d$ and $B^d$, but corresponding to the Skorokhod representation.

It is also straightforward to deduce that the extended sequence $(\tilde{X} ^{d},\tilde{B}^d(\tilde{X}^d),\tilde{W}^{d})_{d}$ is tight in ${\mathscr {G}}\times$ ${\mathscr {G}}\times {\mathbb {R}}$ from which we can deduce by Theorem 4 in [14] that there exists a subsequence, for which we keep the same index, such that

$$\begin{aligned} \left( \tilde{X}^{d},\tilde{B}^d(\tilde{X}^d),\tilde{W}^{d},\int _{0}^{\cdot } \tilde{B}^d(\tilde{X}^d_{s})d\tilde{W}_{s}^{d}\right) _{d}{\ } \end{aligned}$$

converges in distribution to

$$\begin{aligned} \left( \tilde{X},\tilde{B}(\tilde{X}),\tilde{W},\int _{0}^{\cdot }\tilde{B}( \tilde{X}_{s})d\tilde{W}_{s}\right) _{d}, \end{aligned}$$

where $\tilde{W}$ is a Brownian motion. By using one more time the Skorokhod representation theorem, we can assume that all processes are defined on the same probability space which we still denote by $\left( \tilde{\Omega },\tilde{{\mathcal {F}}},\tilde{{\mathbb {P}}}\right)$ and the convergence is in ${\mathscr {G}}\times$ ${\mathscr {G}}\times \mathbb {R\times } {\mathscr {G}}.$ Also, $\tilde{X}^{d}$ satisfies (3.6) and (3.7) on the new space. We will pass to the limit in ${\mathscr {G}}$ in each term of the equation (3.6) for $\tilde{X}^{d}$. The convergence holds immediately for the terms $\tilde{X}_{t}^{d}$ and $\tilde{X}_{0}^{d}$, but we also need to show that both $\int _{0}^{t}\tilde{A}_{s}^{d}( \tilde{X}_{s}^{d})ds$ and $\int _{0}^{t}\tilde{B}_{s}^{d}(\tilde{X} _{s}^{d})d\tilde{W}_{s}^{d}$ converge to $\int _{0}^{t}\tilde{A}_s(\tilde{X}_s)ds$ and $\int _{0}^{t}\tilde{B}_s(\tilde{X}_s)d\tilde{W}_s$, respectively, **in** ${\mathscr {G}}$. For the first integral we use that

3.8 $$\begin{aligned} \begin{aligned} \Vert \tilde{A}_s^d(\tilde{X}_s^d)-\tilde{A}_s(\tilde{X}_s)\Vert _{{\mathscr {G}}} \le \Vert \tilde{A}_s^d(\tilde{X}_s^d) - \tilde{A}_s^d(\tilde{X}_s)\Vert _{{\mathscr {G}}} + \Vert \tilde{A}_s^d(\tilde{X}_s) - \tilde{A}_s(\tilde{X}_s)\Vert _{{\mathscr {G}}}. \end{aligned} \end{aligned}$$

The control of the terms above depends on the initial assumptions, which are different in each of the three cases. We need, therefore, to treat each of these cases separately.

**Case I:** We have from Assumption (*A*1) that

3.9 $$\begin{aligned} \begin{aligned} \Vert \tilde{A}_s^d(\tilde{X}_s^d) - \tilde{A}_s^d(\tilde{X}_s)\Vert _{{\mathscr {G}}} \le C(1 + \Vert \tilde{X}_s^d\Vert _{{\mathscr {F}}_1} + \Vert \tilde{X}_s\Vert _{{\mathscr {F}}_1})\Vert \tilde{X}_s^d - \tilde{X}_s\Vert _{{\mathscr {F}}_0}. \end{aligned} \end{aligned}$$

But we know from Proposition 14 and Proposition 11 that $(\tilde{X}^d)_d$ is tight in ${\mathscr {G}}$ and there is a uniform control for $\displaystyle \int _0^t\Vert \tilde{X}_s^d\Vert _{{\mathscr {F}}_1}ds$. Then

3.10 $$\begin{aligned} \Vert \tilde{X}_s^d - \tilde{X}_s\Vert _{{\mathscr {F}}_0} \xrightarrow {d\rightarrow \infty } 0. \end{aligned}$$

It also holds that

3.11 $$\begin{aligned} \begin{aligned} \Vert \tilde{A}_s^d(\tilde{X}_s) - \tilde{A}_s(\tilde{X}_s)\Vert _{{\mathscr {G}}} \xrightarrow {d\rightarrow \infty } 0 \end{aligned} \end{aligned}$$

since $\tilde{A}^d$ is the projection of $\tilde{A}$ through the operator $T_d$. More precisely, in the first case we have that

$$\begin{aligned} \begin{aligned} \int _{0}^{t}\Vert \tilde{A}_s^{d}(\tilde{X}_{s})-\tilde{A}_s(\tilde{X}_{s})\Vert _{{\mathscr {G}}}ds&\le \int _{0}^{t}\Vert \tilde{A}_s^{d}(\tilde{X}_{s})\Vert _{{\mathscr {G}}}+\Vert \tilde{A}_s(\tilde{X}_{s})\Vert _{{\mathscr {G}}}ds\\&\le 2\int _{0}^{t}\Vert \tilde{A}_s(\tilde{X}_{s})\Vert _{{\mathscr {G}}}ds \\&\le 2C_1\left( \int _{0}^{t}\left\| \tilde{X}_{s}\right\| _{{\mathscr {F}}_{0}}^{\gamma ^1}ds+C_2\int _{0}^{t}\left\| \tilde{X}_{s}\right\| _{{\mathscr {F}}_{1}}^{2}ds+1\right) \end{aligned} \end{aligned}$$

which holds due to the Assumption (*A*1) (with $\gamma ^2=2$) for $\tilde{A}^d$ and Fatou’s lemma. Then since

$$\begin{aligned} \lim _{d\rightarrow \infty }\Vert \tilde{A}_s^{d}(\tilde{X}_{s})-\tilde{A}_s(\tilde{X}_{s})\Vert _{{\mathscr {G}}}=0 \end{aligned}$$

it follows by the dominated convergence theorem that

$$\begin{aligned} \displaystyle \lim _{d\rightarrow \infty }\int _{0}^{t}\Vert \tilde{A}_s^{d}(\tilde{X}_{s})-\tilde{A}_s(\tilde{X}_{s})\Vert _{{\mathscr {G}}}ds=0 \end{aligned}$$

${\mathbb {P}}$-almost surely and therefore also in probability. Next we also have that

$$\begin{aligned} \begin{aligned} \Vert \tilde{A}_s^{d}(\tilde{X}_{s}^{d})-\tilde{A}_s^{d}(\tilde{X}_{s})\Vert _{{\mathscr {G}}}&\le C\left( 1+\Vert \tilde{X}_{s}^{d}\Vert _{{\mathscr {F}}_{1}} + \Vert \tilde{X}_{s}\Vert _{{\mathscr {F}}_{1}}\right) \Vert \tilde{X}_{s}^{d}-\tilde{X}_{s}\Vert _{{\mathscr {F}}_{0}} \end{aligned} \end{aligned}$$

based on the same assumption (*A*1) and Fatou’s lemma. Hence

$$\begin{aligned}  &   \left( \int _{0}^{t}\Vert \tilde{A}_s^{d}(\tilde{X}_{s}^{d})-\tilde{A}_s^{d}(\tilde{X}_{s})\Vert _{{\mathscr {G}} }ds\right) ^{2}\le 4C^2 \int _{0}^{t}\left( 1+ \Vert \tilde{X}_{s}^{d}\Vert _{{\mathscr {F}}_{1}}^2+ \Vert \tilde{X}_{s}\Vert _{{\mathscr {F}}_{1}}^2\right) \\  &   \quad ds\int _{0}^{t}\Vert \tilde{X}_{s}^{d} - \tilde{X}_{s}\Vert _{{\mathscr {F}}_{0}}^{2}ds \end{aligned}$$

and therefore

$$\begin{aligned} \begin{aligned}&\lim _{d\rightarrow \infty }\left( \int _{0}^{t}\Vert \tilde{A}_s^{d}(\tilde{X}_{s}^{d})-\tilde{A}_s^{d}(\tilde{X}_{s})\Vert _{{\mathscr {G}}}ds\right) ^{2} \le \displaystyle \limsup _{d\rightarrow \infty }\displaystyle \int _0^t\left( 1+ \Vert \tilde{X}_{s}^{d}\Vert _{{\mathscr {F}}_{1}}^2+ \Vert \tilde{X}_{s}\Vert _{{\mathscr {F}}_{1}}^2\right) \\&\quad ds \lim _{d\rightarrow \infty }\int _{0}^{t}\Vert \tilde{X}_{s}^{d}-\tilde{X}_{s}\Vert _{{\mathscr {F}}_{0}}^{2}ds \\  &= 0 .\\ \end{aligned} \end{aligned}$$

Note that the first term is uniformly bounded in *d* from Proposition 11 hence the limit is indeed 0.

**Case II:** This follows using exactly the same arguments, since condition (2.19) is the same. We have, more precisely,

$$\begin{aligned} \begin{aligned} \int _{0}^{t}\Vert \tilde{A}_s^{d}(\tilde{X}_{s})-\tilde{A}_s(\tilde{X}_{s})\Vert _{{\mathscr {G}}}ds&\le 2C_1 \int _{0}^{t}\left\| \tilde{X}_{s}\right\| _{{\mathscr {F}}_{0}}^{\gamma ^1}ds \end{aligned} \end{aligned}$$

and

$$\begin{aligned} \begin{aligned} \Vert \tilde{A}_s^{d}(\tilde{X}_{s}^{d})-\tilde{A}_s^{d}(\tilde{X}_{s})\Vert _{{\mathscr {G}}}&\le C\left( 1+\Vert \tilde{X}_{s}^{d}\Vert _{{\mathscr {F}}_{1}} + \Vert \tilde{X}_{s}\Vert _{{\mathscr {F}}_{1}}\right) \Vert \tilde{X}_{s}^{d}-\tilde{X}_{s}\Vert _{{\mathscr {F}}_{0}} \end{aligned} \end{aligned}$$

and the limit when $d\rightarrow \infty$ is zero as before.

**Case III:** From Proposition 14 and Proposition 11,

3.12 $$\begin{aligned} \Vert \tilde{X}_s^d - \tilde{X}_s\Vert _{{\mathscr {F}}_1} \xrightarrow {d\rightarrow \infty } 0. \end{aligned}$$

Also

3.13 $$\begin{aligned} \begin{aligned} \Vert \tilde{A}_s^d(\tilde{X}_s) - \tilde{A}_s(\tilde{X}_s)\Vert _{{\mathscr {G}}} \xrightarrow {d\rightarrow \infty } 0 \end{aligned} \end{aligned}$$

by the same arguments as before. Then

$$\begin{aligned} \begin{aligned} \int _{0}^{t}\Vert \tilde{A}_s^{d}(\tilde{X}_{s})-\tilde{A}_s(\tilde{X}_{s})\Vert _{{\mathscr {G}}}ds\le &   2C_2 \int _{0}^{t}\left\| \tilde{X}_{s}\right\| _{{\mathscr {F}}_{1}}^{\gamma ^2}ds. \end{aligned} \end{aligned}$$

Then by the dominated convergence theorem

$$\begin{aligned} \displaystyle \lim _{d\rightarrow \infty }\int _{0}^{t}\Vert \tilde{A}_s^{d}(\tilde{X}_{s})-\tilde{A}_s(\tilde{X}_{s})\Vert _{{\mathscr {G}}}ds=0 \end{aligned}$$

${\mathbb {P}}$-almost surely and therefore also in probability. Next we have that

3.14 $$\begin{aligned} \begin{aligned} \Vert \tilde{A}_s^{d}(\tilde{X}_{s}^{d})-\tilde{A}_s^{d}(\tilde{X}_{s})\Vert _{{\mathscr {G}}}&\le C\left( 1+\Vert \tilde{X}_{s}^{d}\Vert _{{\mathscr {F}}_{1}} + \Vert \tilde{X}_{s}\Vert _{{\mathscr {F}}_{1}}\right) \Vert \tilde{X}_{s}^{d}-\tilde{X}_{s}\Vert _{{\mathscr {F}}_{1}} \end{aligned} \end{aligned}$$

based on the assumption (*A*3) and Fatou’s lemma. Hence

$$\begin{aligned} \left( \int _{0}^{t}\Vert \tilde{A}_s^{d}(\tilde{X}_{s}^{d})-\tilde{A}_s^{d}(\tilde{X}_{s})\Vert _{{\mathscr {G}} }ds\right) ^{2}\le &   4C^2 \int _{0}^{t}\left( 1+ \Vert \tilde{X}_{s}^{d}\Vert _{{\mathscr {F}}_{1}}^2+ \Vert \tilde{X}_{s}\Vert _{{\mathscr {F}}_{1}}^2\right) ds\\  &   \,\int _{0}^{t}\Vert \tilde{X}_{s}^{d} - \tilde{X}_{s}\Vert _{{\mathscr {F}}_{1}}^{2}ds \end{aligned}$$

and

$$\begin{aligned} \begin{aligned} \lim _{d\rightarrow \infty }\left( \int _{0}^{t}\Vert \tilde{A}_s^{d}(\tilde{X}_{s}^{d}) - \tilde{A}_s^{d}(\tilde{X}_{s})\Vert _{{\mathscr {G}}}ds\right) ^{2} = 0 \\ \end{aligned} \end{aligned}$$

as before. We now justify the convergence of the stochastic integral. Let

3.15 $$\begin{aligned}  &   \begin{aligned} a_d(t) := \displaystyle \int _0^t \tilde{B}_s(\tilde{X}_s)d\tilde{W}_s^d - \displaystyle \int _0^t \tilde{B}_s(\tilde{X}_s)d\tilde{W}_s \end{aligned} \end{aligned}$$

3.16 $$\begin{aligned}  &   \begin{aligned} b_d(t) := \displaystyle \int _0^t \tilde{B}_s^d(\tilde{X}_s^d)d\tilde{W}_s^d - \displaystyle \int _0^t \tilde{B}_s(\tilde{X}_s)d\tilde{W}_s^d. \end{aligned} \end{aligned}$$

We need to show that $a_d(t) + b_d(t) \xrightarrow {d\rightarrow \infty } 0$ in probability. We already know that $a_d(t) \xrightarrow {d\rightarrow \infty } 0$ almost surely in ${\mathscr {G}}$ (hence in probability). By Lemma 10 below

3.17 $$\begin{aligned} \displaystyle \int _0^t \Vert \tilde{B}_s^d(\tilde{X_s^d}) - \tilde{B}_s(\tilde{X}_s)\Vert _{{\mathscr {G}}}^2ds \xrightarrow {d\rightarrow \infty }0 \end{aligned}$$

in probability. Due to this limit and using Proposition 18 we can write

3.18 $$\begin{aligned} {\mathbb {P}}\left( \displaystyle \sup _{s\in [0,t]}\Vert b_d(s)\Vert _{{\mathscr {G}}}> \epsilon \right) \le \frac{\eta }{\epsilon ^2} + {\mathbb {P}}\left( \displaystyle \int _0^t \Vert \tilde{B}_s^d(\tilde{X_s^d}) - \tilde{B}_s(\tilde{X}_s)\Vert _{{\mathscr {G}}}^2 ds> \eta \right) .\nonumber \\ \end{aligned}$$

Then we can choose $\eta :=\frac{\delta }{2\epsilon ^2}$ and *d* sufficiently large such that

3.19 $$\begin{aligned} {\mathbb {P}}\left( \displaystyle \int _0^t \Vert \tilde{B}_s^d(\tilde{X_s^d}) - \tilde{B}_s(\tilde{X}_s)\Vert _{{\mathscr {G}}}^2ds > \frac{\delta }{2\epsilon ^2} \right) < \frac{\delta }{2} \end{aligned}$$

which gives that also $b_d(t) \xrightarrow {d\rightarrow \infty } 0$ in probability.

### Lemma 10

We have

3.20 $$\begin{aligned} \displaystyle \int _0^t \Vert \tilde{B}_s^d(\tilde{X_s^d}) - \tilde{B}_s(\tilde{X}_s)\Vert _{{\mathscr {G}}}^2ds \xrightarrow {d\rightarrow \infty }0 \end{aligned}$$

in probability.

### Proof

**Case I**: We can write

$$\begin{aligned} \begin{aligned}&\Vert \tilde{B}_s^d(\tilde{X_s^d}) - \tilde{B}_s(\tilde{X}_s)\Vert _{{\mathscr {G}}} \\&\quad \le \Vert \tilde{X}_s^d\Vert _{{\mathscr {F}}_0}\left( \Vert \tilde{X}_s^d - \tilde{X}_s\Vert _{{\mathscr {G}}} \right) + \left( \Vert X_s^d\Vert _{{\mathscr {F}}_0} - \Vert \tilde{X}_s\Vert _{{\mathscr {F}}_0}\right) \Vert \tilde{X}_s\Vert _{{\mathscr {G}}} \\&\quad \le \displaystyle \sup _{s}\Vert \tilde{X}_s^d\Vert _{{\mathscr {F}}_0}\left( \Vert \tilde{X}_s^d - \tilde{X}_s\Vert _{{\mathscr {G}}} \right) + \displaystyle \sup _{s}\Vert \tilde{X}_s\Vert _{{\mathscr {G}}}\left( \Vert \tilde{X}_s^d\Vert _{{\mathscr {F}}_0} - \Vert \tilde{X}_s\Vert _{{\mathscr {F}}_0}\right) . \end{aligned} \end{aligned}$$

We will prove that each of these two terms converges to zero in probability. Let

$$\begin{aligned}  &   c_d(t):= \displaystyle \sup _{s\in [0,t]}\Vert \tilde{X}_s^d\Vert _{{\mathscr {F}}_0}\displaystyle \int _0^t \Vert \tilde{X}_s^d - \tilde{X}_s\Vert _{{\mathscr {G}}} ds \\  &   \quad e_d(t):= \displaystyle \sup _{s\in [0,t]}\Vert \tilde{X}_s\Vert _{{\mathscr {G}}}\displaystyle \int _0^t\Vert \tilde{X}_s^d -\tilde{X}_s\Vert _{{\mathscr {F}}_0}ds. \end{aligned}$$

Note that since $X_s^d \rightarrow X_s$ in ${\mathscr {G}}$ and

$$\begin{aligned} \Vert \tilde{X}_s^d-\tilde{X}_s\Vert _{{\mathscr {G}}} \le C\displaystyle \sup _{s}\Vert \tilde{X}_s\Vert _{{\mathscr {G}}} \le C\displaystyle \sup _{s}\Vert \tilde{X}_s\Vert _{{\mathscr {F}}_0} \end{aligned}$$

which is bounded in probability by Proposition 11 and the Fatou lemma, we have

$$\begin{aligned} \displaystyle \lim _{d\rightarrow \infty } \displaystyle \int _0^t\Vert \tilde{X}_s^d-\tilde{X}_s\Vert _{{\mathscr {G}}} ds = 0 \end{aligned}$$

in probability. We can choose *R* such that

3.21 $$\begin{aligned} {\mathbb {P}}\left( \displaystyle \sup _{s\in [0,T]}\Vert \tilde{X}_s^d\Vert _{{\mathscr {F}}_0} >R\right) < \frac{\delta }{2} \end{aligned}$$

and *d* such that

3.22 $$\begin{aligned} {\mathbb {P}}\left( \displaystyle \int _0^t\Vert \tilde{X}_s^d-\tilde{X}_s\Vert _{{\mathscr {G}}}ds > \frac{\epsilon }{R} \right) < \frac{\delta }{2}. \end{aligned}$$

It follows that

$$\begin{aligned} \begin{aligned} {\mathbb {P}}\left( c_d(t) \ge \epsilon \right)&= {\mathbb {P}}\left( c_d(t) \ge \epsilon , \displaystyle \sup _{s\in [0,T]}\Vert \tilde{X}_s^d\Vert _{{\mathscr {F}}_0}>R\right) \\&\quad + {\mathbb {P}}\left( c_d(t) \ge \epsilon , \displaystyle \sup _{s\in [0,T]}\Vert \tilde{X}_s^d\Vert _{{\mathscr {F}}_0}< R \right) \\&\le \frac{\delta }{2} + {\mathbb {P}}\left( c_d(t) \ge \epsilon , \displaystyle \sup _{s\in [0,T]}\Vert \tilde{X}_s^d\Vert _{{\mathscr {F}}_0}>R, \displaystyle \int _0^t\Vert \tilde{X}_s^d-\tilde{X}_s\Vert _{{\mathscr {G}}}ds> \frac{\epsilon }{R}\right) \\&\quad + {\mathbb {P}}\left( c_d(t) \ge \epsilon , \displaystyle \sup _{s\in [0,T]}\Vert \tilde{X}_s^d\Vert _{{\mathscr {F}}_0}>R, \displaystyle \int _0^t\Vert \tilde{X}_s^d-\tilde{X}_s\Vert _{{\mathscr {G}}}ds < \frac{\epsilon }{R}\right) \\&\le \frac{\delta }{2} + {\mathbb {P}}\left( \displaystyle \int _0^t\Vert \tilde{X}_s^d-\tilde{X}_s\Vert _{{\mathscr {G}}}ds > \frac{\epsilon }{R} \right) \\&\le \frac{\delta }{2} + \frac{\delta }{2} = \delta . \end{aligned} \end{aligned}$$

For showing convergence (in probability) of $e_d(t)$ we proceed in a similar way, using the fact that $\displaystyle \sup _{s}\Vert \tilde{X}_s\Vert _{{\mathscr {G}}}$ is bounded in probability and that

$$\begin{aligned} \begin{aligned} \displaystyle \int _0^t\Vert \tilde{X}_s^d-\tilde{X}_s\Vert _{{\mathscr {F}}_0}ds&\le \displaystyle \int _0^t\Vert \tilde{X}_s^d-\tilde{X}_s\Vert _{{\mathscr {F}}_1}^{\theta ^{\star }}\Vert \tilde{X}_s^d-\tilde{X}_s\Vert _{{\mathscr {G}}}^{1-\theta ^{\star }}ds \\&\le \left( \displaystyle \int _0^t\Vert \tilde{X}_s^d-\tilde{X}_s\Vert _{{\mathscr {F}}_1}ds \right) ^{1/2} \left( \displaystyle \int _0^t \Vert \tilde{X}_s^d-\tilde{X}_s\Vert _{{\mathscr {G}}}^{1-\theta ^{\star }}\right) ^{1/2} \end{aligned} \end{aligned}$$

and the last term converges to zero in probability, while the integral of the ${\mathscr {F}}_1$ norm of the difference is bounded in probability. The control in (3.21) and (3.22) holds also in Case II and Case III, by the same arguments.

### Uniform control

#### Proposition 11

The approximating sequence $(X_t^d)_d$ is ${\mathbb {P}}$-a.s. uniformly controlled in ${\mathscr {F}}_0$ in the uniform norm, and in ${\mathscr {F}}_1$ in $L^2$ sense. That is for any $\epsilon _1,\epsilon _2 >0$ there exists ${\mathcal {K}}_1,{\mathcal {K}}_2 >0$ such that

3.23 $$\begin{aligned} \displaystyle \sup _d {\mathbb {P}}\left( \displaystyle \sup _t \Vert X_t^d\Vert _{{\mathscr {F}}_0}^2 \ge {\mathcal {K}}_1\right) \le \epsilon _1 \end{aligned}$$

and

3.24 $$\begin{aligned} \displaystyle \sup _d {\mathbb {P}}\left( \displaystyle \int _0^T \Vert X_s^d\Vert _{{\mathscr {F}}_1}^2ds \ge {\mathcal {K}}_2\right) \le \epsilon _2. \end{aligned}$$

#### Proof

We first prove (3.23) and then deduce (3.24) from it, when the space ${\mathscr {F}}_1$ exists. In order to prove (3.23), we first show that for any $\tilde{\epsilon }>0$ there exists $\tilde{{\mathcal {K}}}$ such that

3.25 $$\begin{aligned} \displaystyle \sup _d {\mathbb {P}}\left( \displaystyle \sup _t \log (C+\Vert X_t^d\Vert _{{\mathscr {F}}_0}^2 )\ge \tilde{{\mathcal {K}}}\right) \le \tilde{\epsilon } \end{aligned}$$

and then the conclusion will follow. By the Itô formula we have

3.26 $$\begin{aligned} \begin{aligned} d\Vert X_t^d\Vert _{{\mathscr {F}}_0}^2&= \tilde{A}_t(X_t^d)dt + \tilde{B}_t(X_t^d)dW_t^d \end{aligned} \end{aligned}$$

where

3.27 $$\begin{aligned} \tilde{A}_t(X_t^d):= \left( 2{}_{\mathscr {D}}\langle X_t^d, A_t^d(X_t^d)\rangle _{{\mathscr {G}}} + \Vert B_t(X_t^d)\Vert _{L_2({\mathscr {U}}, {\mathscr {F}}_0)}^2\right) \end{aligned}$$

and

3.28 $$\begin{aligned} \tilde{B}_t(X_t^d)dW_t^d:= 2\langle X_t^d, B_t(X_t^d)dW_t\rangle _{{\mathscr {F}}_0}. \end{aligned}$$

Then

3.29 $$\begin{aligned} \begin{aligned}&d\log \left( C+ \Vert X_t^d\Vert _{{\mathscr {F}}_0}^2 \right) = \frac{1}{C+ \Vert X_t^d\Vert _{{\mathscr {F}}_0}^2}\left( \tilde{A}_t^d(X_t^d)dt + \tilde{B}_t^d(X_t^d)dW_t^d \right) \\&\qquad - \frac{1}{2(C+ \Vert X_t^d\Vert _{{\mathscr {F}}_0}^2)^2}\tilde{B}_t ^d(X_t^d)^2dt \\&\quad = \frac{1}{C+ \Vert X_t^d\Vert _{{\mathscr {F}}_0}^2}\tilde{A}_t^d(X_t^d)dt - \frac{1-\epsilon }{2(C+ \Vert X_t^d\Vert _{{\mathscr {F}}_0}^2)^2}\tilde{B}_t ^d(X_t^d)^2dt \\&\qquad + \frac{1}{C+ \Vert X_t^d\Vert _{{\mathscr {F}}_0}^2}\tilde{B}_t^d(X_t^d)dW_t^d - \frac{\epsilon }{2(C+ \Vert X_t^d\Vert _{{\mathscr {F}}_0}^2)^2}\tilde{B}_t ^d(X_t^d)^2dt. \end{aligned} \end{aligned}$$

If we choose

3.30 $$\begin{aligned} M_t:=\displaystyle \int _0^t\tilde{B}_s^d(X_s^d)dW_s \end{aligned}$$

then

3.31 $$\begin{aligned} \langle M \rangle _t = \displaystyle \int _0^t\tilde{B}_s^d(X_s)^2 ds. \end{aligned}$$

As suggested above, we will now need to split the proof in three distinct cases, depending on the specific form of the diffusion term *B*:

**Case I:** The diffusion term is given by

3.32 $$\begin{aligned} B_t^d(X_t^d) = \theta \Vert X_t^d\Vert _{{\mathscr {F}}_0}^{\alpha _0}X_t^d. \end{aligned}$$

In this case

3.33 $$\begin{aligned}  &   \begin{aligned} \Vert B_t^d(X_t^d)\Vert _{L_2({\mathscr {U}}, {\mathscr {F}}_0)}^2&= \theta ^2\Vert X_t^d\Vert _{{\mathscr {F}}_0}^{2\alpha _0+2} \end{aligned} \end{aligned}$$

3.34 $$\begin{aligned}  &   \tilde{B}_t^d(X_t^d)^2:=(2\langle X_t^d, B_t^d(X_t^d)\rangle )^2 = 4\theta ^2 \Vert X_t^d\Vert _{{\mathscr {F}}_0}^{2\alpha _0+4} \end{aligned}$$

and

3.35 $$\begin{aligned} \begin{aligned} \tilde{A}_t^d(X_t^d)&=2{}_{\mathscr {D}}\langle X_t^d, A_t^d(X_t^d)\rangle _{{\mathscr {G}}} + \Vert B_t(X_t^d)\Vert _{L_2({\mathscr {U}}, {\mathscr {F}}_0)}^2 \\&= 2\langle X_t^d, A_t^d(X_t^d)\rangle _{{\mathscr {F}}_0} + \theta ^2\Vert X_t^d\Vert _{{\mathscr {F}}_0}^{2\alpha _0+2}\\&\le 2C_1\Vert X_t^d\Vert _{{\mathscr {F}}_0}^{\gamma _1} - 2C_2\Vert X_t^d\Vert _{{\mathscr {F}}_1}^{\gamma _2}+ \theta ^2\Vert X_t^d\Vert _{{\mathscr {F}}_0}^{2\alpha _0+2} \end{aligned} \end{aligned}$$

due to conditions (2.2) and (2.16). Let us define the semimartingale

3.36 $$\begin{aligned} dM_t := 2\theta _1\langle X_t^d, \Vert X_t^d\Vert _{{\mathscr {F}}_0}^{\alpha _1}X_t^d\rangle dW_t^d. \end{aligned}$$

Then

3.37 $$\begin{aligned} \begin{aligned} d\Vert X_t^d\Vert _{{\mathscr {F}}_0}^2 + \tilde{C}_2\Vert X_t^d\Vert _{{\mathscr {F}}_1}^{\gamma _2}dt \le G \left( \Vert X_t^d\Vert _{{\mathscr {F}}_0}\right) dt + dM_t \end{aligned} \end{aligned}$$

where $\tilde{C}_2 >0$ and it depends on $C_2$ and^3^

3.38 $$\begin{aligned} G \left( \Vert X_t^d\Vert _{{\mathscr {F}}_0}\right) := \tilde{C}_1\Vert X_t^d\Vert _{{\mathscr {F}}_0}^{\gamma _1} + \theta ^2\Vert X_t^d\Vert _{{\mathscr {F}}_0}^{2\alpha _0+2}. \end{aligned}$$

But then

3.39 $$\begin{aligned} \begin{aligned} d\Vert X_t^d\Vert _{{\mathscr {F}}_0}^2 \le G(\Vert X_t^d\Vert _{{\mathscr {F}}_0})dt + \tilde{B}_t(X_t^d)dW_t \end{aligned} \end{aligned}$$

and for C>0 and

3.40 $$\begin{aligned} \tilde{C}(X_t^d) := C+ \Vert X_t^d\Vert _{{\mathscr {F}}_0}^2 \end{aligned}$$

we can write

$$\begin{aligned}&d \log (C+\Vert X_t^d\Vert _{{\mathscr {F}}_0}^2) \le \frac{1}{\tilde{C}(X_t^d)^2}\left( \tilde{C}(X_t^d)G(\Vert X_t^d\Vert _{{\mathscr {F}}_0}) - \frac{1-\epsilon }{2}\tilde{B}_t^d(X_t^d)^2\right) dt \\&\qquad + dM_t - \frac{\epsilon }{2}d\langle M\rangle _t\\&\quad = \frac{1}{\tilde{C}(X_t^d)^2}\left( \tilde{C}(X_t^d)(\tilde{C}_1\Vert X_t^d\Vert _ {{\mathscr {F}}_0}^{\gamma _1}+\Vert B_t^d(X_t^d)\Vert _{L_2({\mathscr {U}}, {\mathscr {F}}_0)}^2 ) - \frac{1-\epsilon }{2}\tilde{B}_t^d(X_t^d)^2\right) dt \\&\qquad + dM_t - \frac{\epsilon }{2}d\langle M\rangle _t \\&\quad = \frac{1}{(C+ \Vert X_t^d\Vert _{{\mathscr {F}}_0}^2)^2} \left( (C+ \Vert X_t^d\Vert _{{\mathscr {F}}_0}^2)(\tilde{C}_1\Vert X_t^d\Vert _{{\mathscr {F}}_0}^{\gamma _1} + \theta ^2\Vert X_t^d\Vert _{{\mathscr {F}}_0}^{2\alpha _0+2})\right. \\&\qquad \left. - \frac{1-\epsilon }{2}4\theta ^2\Vert X_t^d\Vert _{{\mathscr {F}}_0}^{2\alpha _0+4}\right) dt + dM_t - \frac{\epsilon }{2}d\langle M\rangle _t \\&\quad = \frac{1}{(C+ \Vert X_t^d\Vert _{{\mathscr {F}}_0}^2)^2} \left( \tilde{C}_{11}\Vert X_t^d\Vert _{{\mathscr {F}}_0}^{\gamma _1} + C\theta ^2 \Vert X_t^d\Vert _{{\mathscr {F}}_0}^{2\alpha _0+2} + \tilde{C}_1\Vert X_t^d\Vert _{{\mathscr {F}}_0}^{\gamma _1+2} \right. \\&\qquad \left. + \theta ^2\Vert X_t^d\Vert _{{\mathscr {F}}_0}^{2\alpha _0+4} - \frac{1-\epsilon }{2}4\theta ^2\Vert X_t^d\Vert _{{\mathscr {F}}_0}^{2\alpha _0+4}\right) dt + dM_t - \frac{\epsilon }{2}d\langle M\rangle _t \\&\quad = \frac{1}{(C+ \Vert X_t^d\Vert _{{\mathscr {F}}_0}^2)^2} \left( \tilde{C}_1\Vert X_t^d\Vert _{{\mathscr {F}}_0}^{\gamma _1+2} + \tilde{C}_{11}\Vert X_t^d\Vert _{{\mathscr {F}}_0}^{\gamma _1} + C\theta ^2 \Vert X_t^d\Vert _{{\mathscr {F}}_0}^{2\alpha _0+2} \right. \\&\qquad \left. + (2\epsilon -1)\theta ^2\Vert X_t^d\Vert _{{\mathscr {F}}_0}^{2\alpha _0+4}\right) dt + dM_t - \frac{\epsilon }{2}d\langle M\rangle _t. \end{aligned}$$

We want 2ϵ-1<0 and for this reason we choose $\epsilon < \frac{1}{2}$. We know by assumption also that $2\alpha _0>\gamma _1$. Therefore we control any possible blow-up of the drift term, since there exists a positive constant *R* such that

3.41 $$\begin{aligned} \tilde{C}_1\Vert X_t^d\Vert _{{\mathscr {F}}_0}^{\gamma _1+2} + \tilde{C}_{11}\Vert X_t^d\Vert _{{\mathscr {F}}_0}^{\gamma _1} + C\theta ^2 \Vert X_t^d\Vert _{{\mathscr {F}}_0}^{2\alpha _0+2} + (2\epsilon -1)\theta ^2\Vert X_t^d\Vert _{{\mathscr {F}}_0}^{2\alpha _0+4} \le R.\nonumber \\ \end{aligned}$$

Then, as the term on the left hand side of (3.41) has negative leading coefficient, we eventually obtain

3.42 $$\begin{aligned} d\log (C+\Vert X_t^d\Vert _{{\mathscr {F}}_0}^2 )\le k_0 + kt + E(\epsilon ) \end{aligned}$$

with

3.43 $$\begin{aligned} E(\epsilon ) := \sup _{t\ge 0}\left( M_t - \frac{\epsilon }{2}\langle M\rangle _t\right) . \end{aligned}$$

Therefore

3.44 $$\begin{aligned} \displaystyle \sup _{t\in [0,T]}\Vert X_t^d\Vert _{{\mathscr {F}}_0}^2 \le \tilde{E}(\epsilon ) \end{aligned}$$

where

3.45 $$\begin{aligned} \tilde{E}(\epsilon ) = \exp (k_0+kT+E(\epsilon ))-C. \end{aligned}$$

Using Exercise 3.16 from [23] we deduce that

3.46 $$\begin{aligned} {\mathbb {P}}\left( \displaystyle \sup _{t\in [0,T]}\Vert X_t^d\Vert _{{\mathscr {F}}_0}^2 \ge \beta _1\right) < \delta _1. \end{aligned}$$

Note that the random variable on the right hand side of (3.45) is finite, but not integrable. It follows that

3.47 $$\begin{aligned} {\mathbb {P}}(\sup _{t\in [0,T]} (C+\Vert X_t^d\Vert _{{\mathscr {F}}_0})> K) \le {\mathbb {P}}(E(\epsilon ) > \log K - k_0+kT). \end{aligned}$$

Note also that the quantity on the right hand side of the above inequality is independent of the dimension *d*, (as E(ϵ) is *d*-independent and has an exponential distribution), hence

3.48 $$\begin{aligned} \displaystyle \lim _{K\rightarrow \infty } \sup _{d} {\mathbb {P}}(\sup _{t\in [0,T]} {\Vert X_t^d\Vert _{{\mathscr {F}}_0}}> K) \le \displaystyle \lim _{K\rightarrow \infty } {\mathbb {P}}(E(\epsilon ) > \log K - k_0+kT)=0.\nonumber \\ \end{aligned}$$

Now we can proceed with controlling $\tilde{C}_2\int _0^t \Vert X_s^d\Vert _{{\mathscr {F}}_1}^{\gamma _2}ds$. From (3.37) one has

3.49 $$\begin{aligned} \begin{aligned} \tilde{C}_2\displaystyle \int _0^t \Vert X_s^d\Vert _{{\mathscr {F}}_1}^{\gamma _2} \le k_0 - \Vert X_t^d\Vert _{{\mathscr {F}}_0}^2 + \displaystyle \int _0^t G(\Vert X_s^d\Vert _{{\mathscr {F}}_0})ds + \displaystyle \int _0^t \tilde{B}_s^d(X_s^d)dW_s. \end{aligned}\nonumber \\ \end{aligned}$$

Let

3.50 $$\begin{aligned} \begin{aligned} H_t(\Vert X_t^d\Vert _{{\mathscr {F}}_0}) := k_0 - \Vert X_t^d\Vert _{{\mathscr {F}}_0}^2 + \displaystyle \int _0^t G(\Vert X_s^d\Vert _{{\mathscr {F}}_0})ds \end{aligned} \end{aligned}$$

and

3.51 $$\begin{aligned} \tilde{\tilde{B}}_t := \displaystyle \int _0^t \tilde{B}_s^d(X_s^d)dW_s. \end{aligned}$$

Then

3.52 $$\begin{aligned} \tilde{C}_2\displaystyle \int _0^t \Vert X_s^d\Vert _{{\mathscr {F}}_1}^2 \le H_t(\Vert X_t^d\Vert _{{\mathscr {F}}_0})+\frac{\epsilon }{2}\langle \tilde{\tilde{B}}\rangle _t+{\tilde{E}}(\epsilon ) \le C(T,\epsilon )(\sup _{t\in [0,T]}\Vert X_t^d\Vert _{{\mathscr {F}}_0}^{q'})+{\tilde{E}}(\epsilon )\nonumber \\ \end{aligned}$$

with $$ {\tilde{E}}(\epsilon ) := \sup _{t\ge 0}(\tilde{\tilde{B}}_t - \frac{\epsilon }{2}\langle \tilde{\tilde{B}}\rangle _t)$$ and $q^{\prime }$ suitably chosen. This can now be handled as before, which will eventually give

3.53 $$\begin{aligned} {\mathbb {P}}\left( \displaystyle \int _0^t\Vert X_t^d\Vert _{{\mathscr {F}}_1}^{\gamma _2}ds \ge \beta _2\right) < \delta _2 \end{aligned}$$

with $$\tilde{C}_2, \gamma _2 > 0$$.

**Case II:** The diffusion term is given by

3.54 $$\begin{aligned} B_t^d(X_t^d):= \theta \Vert X_t^d\Vert _{{\mathscr {F}}_1}^{\alpha _1}X_t^d. \end{aligned}$$

Then

3.55 $$\begin{aligned}  &   \begin{aligned} \Vert B_t^d(X_t^d)\Vert _{L_2({\mathscr {U}}, {\mathscr {F}}_0)}^2&= \theta ^2\Vert X_t^d\Vert _{{\mathscr {F}}_1}^{2\alpha _1}\Vert X_t^d\Vert _{{\mathscr {F}}_0}^{2} \end{aligned} \end{aligned}$$

3.56 $$\begin{aligned}  &   \quad \tilde{B}_t^d(X_t^d)^2 = 4\theta ^2\Vert X_t^d\Vert _{{\mathscr {F}}_1}^{2\alpha _1}\Vert X_t^d\Vert _{{\mathscr {F}}_0}^{4} \end{aligned}$$

and

3.57 $$\begin{aligned} \begin{aligned} \tilde{A}_t^d(X^d)&=2{}_{\mathscr {D}}\langle X_t^d, A_t^d(X_t^d)\rangle _{{\mathscr {G}}} + \Vert B_t(X_t^d)\Vert _{L_2({\mathscr {U}}, {\mathscr {F}}_0)}^2 \\&= 2\langle X_t^d, A_t^d(X_t^d)\rangle _{{\mathscr {F}}_0} + \theta ^2\Vert X_t^d\Vert _{{\mathscr {F}}_1}^{2\alpha _1}\Vert X_t^d\Vert _{{\mathscr {F}}_0}^{2}\\&\le 2C_1\Vert X_t^d\Vert _{{\mathscr {F}}_0}^{\gamma _1} - 2C_2\Vert X_t^d\Vert _{{\mathscr {F}}_1}^{\gamma _2}+ \theta ^2\Vert X_t^d\Vert _{{\mathscr {F}}_1}^{2\alpha _1}\Vert X_t^d\Vert _{{\mathscr {F}}_0}^{2}. \end{aligned} \end{aligned}$$

We have, as before,

3.58 $$\begin{aligned} \begin{aligned} d\Vert X_t^d\Vert _{{\mathscr {F}}_0}^2&= \left( 2{}_{\mathscr {D}}\langle X_t^d, A_t^d(X_t^d)\rangle _{{\mathscr {G}}} + \Vert B_t^d(X_t^d)\Vert _{L_2({\mathscr {U}}, {\mathscr {F}}_0)}^2\right) dt\\&\quad + 2\langle X_t^d, B_t^d(X_t^d)dW_t\rangle _{{\mathscr {F}}_0} \end{aligned} \end{aligned}$$

and then

$$\begin{aligned}&d \log \Vert X_t^d\Vert _{{\mathscr {F}}_0}^2 =\frac{1}{\Vert X_t^d\Vert _{{\mathscr {F}}_0}^2} \left( 2{}_{\mathscr {D}}\langle X_t^d, A_t^d(X_t^d)\rangle _{{\mathscr {G}}} + \Vert B_t^d(X_t^d)\Vert _{L_2({\mathscr {U}}, {\mathscr {F}}_0)}^2\right) dt\nonumber \\&\quad + \frac{2}{\Vert X_t^d\Vert _{{\mathscr {F}}_0}^2}\langle X_t^d, B_t^d(X_t^d)dW_t\rangle _{{\mathscr {F}}_0} -\frac{2}{\Vert X_t^d\Vert _{{\mathscr {F}}_0}^4}\langle X_t^d, B_t^d(X_t^d)\rangle _{{\mathscr {F}}_0}^2dt \nonumber \\&\quad - \frac{2\epsilon }{\Vert X_t^d\Vert _{{\mathscr {F}}_0}^4}\langle X_t^d, B_t^d(X_t^d)\rangle _{{\mathscr {F}}_0}^2dt + \frac{2\epsilon }{\Vert X_t^d\Vert _{{\mathscr {F}}_0}^4}\langle X_t^d, B_t^d(X_t^d)\rangle _{{\mathscr {F}}_0}^2dt \nonumber \\&= \frac{1}{\Vert X_t^d\Vert _{{\mathscr {F}}_0}^2} \left( 2{}_{\mathscr {D}}\langle X_t^d, A_t^d(X_t^d)\rangle _{{\mathscr {G}}} + \Vert B_t^d(X_t^d)\Vert _{L_2({\mathscr {U}}, {\mathscr {F}}_0)}^2\right) dt \nonumber \\&\quad - \frac{2(1-\epsilon )}{\Vert X_t^d\Vert _{{\mathscr {F}}_0}^4}\langle X_t^d, B_t^d(X_t^d)\rangle _{{\mathscr {F}}_0}^2dt + dN_t - \frac{\epsilon }{2}d\langle N \rangle _t \end{aligned}$$

where

$$\begin{aligned} N_t := \displaystyle \int _0^t \frac{2}{\Vert X_s^d\Vert _{{\mathscr {F}}_0}^2}\langle X_s^d, B_s^d(X_s^d) \rangle _{{\mathscr {F}}_0} dW_s. \end{aligned}$$

The drift above is given by

$$\begin{aligned} p_1:= &   \frac{2}{\Vert X_t^d\Vert _{{\mathscr {F}}_0}^4} \left( \Vert X_t^d\Vert _{{\mathscr {F}}_0}^2 {}_{\mathscr {D}}\langle X_t^d, A_t^d(X_t^d)\rangle _{{\mathscr {G}}} \right. \\  &   \, \left. + \frac{1}{2}\Vert X_t^d\Vert _{{\mathscr {F}}_0}^2\Vert B_t^d(X_t^d)\Vert _{L_2({\mathscr {U}}, {\mathscr {F}}_0)}^2 - (1-\epsilon )\langle X_t^d, B_t^d(X_t^d)\rangle _{{\mathscr {F}}_0}^2 \right) \end{aligned}$$

and by condition (2.16) we have

$$\begin{aligned} \begin{aligned}&\frac{2}{\Vert X_t^d\Vert _{{\mathscr {F}}_0}^4} \left( \Vert X_t^d\Vert _{{\mathscr {F}}_0}^2 \langle X_t^d, A_t^d(X_t^d)\rangle _{{\mathscr {F}}_0} \right. \\&\qquad \left. + \frac{1}{2}\Vert X_t^d\Vert _{{\mathscr {F}}_0}^2\Vert B_t^d(X_t^d)\Vert _{L_2({\mathscr {U}}, {\mathscr {F}}_0)}^2 - (1-\epsilon )\langle X_t^d, B_t^d(X_t^d)\rangle _{{\mathscr {F}}_0}^2 \right) \\&\quad \le \frac{2}{\Vert X_t^d\Vert _{{\mathscr {F}}_0}^4}\big ( C_1\Vert X_t^d\Vert _{{\mathscr {F}}_0}^{\gamma _1+2}-C_2\Vert X_t^d\Vert _{{\mathscr {F}}_0}^2\Vert X_t^d\Vert _{{\mathscr {F}}_1}^2 \\&\qquad +\frac{1}{2}\theta ^2\Vert X_t^d\Vert _{{\mathscr {F}}_1}^{2\alpha _1}\Vert X_t^d\Vert _{{\mathscr {F}}_0}^4 - (1-\epsilon ) \theta ^2\Vert X_t^d\Vert _{{\mathscr {F}}_1}^{2\alpha _1}\Vert X_t^d\Vert _{{\mathscr {F}}_0}^4\big ) \\&\quad \le 2\left( \left( C_1+ \left( \epsilon - \frac{1}{2} \right) \theta ^2\right) \right) \Vert X_t^d\Vert _{{\mathscr {F}}_1}^{2\alpha _1} \end{aligned} \end{aligned}$$

and we can choose $\theta = \frac{2C_1}{1/2- \epsilon }$. This implies that

$$\begin{aligned} d \log \Vert X_t^d\Vert _{{\mathscr {F}}_0} \le dN_t - \frac{\epsilon }{2}d\langle N \rangle _t. \end{aligned}$$

Since we choose $\gamma _1-2<2\alpha _1$, we have

3.59 $$\begin{aligned}  &   2C_1\Vert X_t^d\Vert _{{\mathscr {F}}_0}^{\gamma _1-2} + \left( C_1 + \left( \epsilon - \frac{1}{2}\right) \theta ^2\right) \Vert X_t^d\Vert _{{\mathscr {F}}_1}^{2\alpha _1}\nonumber \\  &   \quad \le 2C_1\Vert X_t^d\Vert _{{\mathscr {F}}_1}^{\gamma _1-2} + \left( C_1 + \left( \epsilon -\frac{1}{2}\right) \theta ^2\right) \Vert X_t^d\Vert _{{\mathscr {F}}_1}^{2\alpha _1} \end{aligned}$$

due to the fact that ${\mathscr {F}}_1 \hookrightarrow {\mathscr {F}}_0$ (and we are in a *d*-dimensional space), which is a polynomial in $\Vert X_t^d\Vert _{{\mathscr {F}}_0}$ with negative highest coefficient which is bounded (as before). So we have

3.60 $$\begin{aligned} \begin{aligned} p_1 \le 2C_1\Vert X_t^d\Vert _{{\mathscr {F}}_0}^{\gamma _1-2} + \left( C_1 + \left( \epsilon - \frac{1}{2}\right) \theta ^2\right) \Vert X_t^d\Vert _{{\mathscr {F}}_0}^{2\alpha _1} dt \end{aligned} \end{aligned}$$

and after replacing this in (3.58) and using the same argument as per Revuz & Yor as before we obtain

3.61 $$\begin{aligned} {\mathbb {P}}\left( \displaystyle \sup _{t\in [0,T]}\Vert X_t^d\Vert _{{\mathscr {F}}_0}^2 \ge \beta _{11}\right) < \delta _{11}. \end{aligned}$$

Now in order to obtain

3.62 $$\begin{aligned} {\mathbb {P}}\left( \displaystyle \sup _{t\in [0,T]}\displaystyle \int _0^t\Vert X_s^d\Vert _{{\mathscr {F}}_1}^{2\alpha _1}ds \ge \beta _{22}\right) < \delta _{22} \end{aligned}$$

the arguments are similar to those from the previous section (viscous case). Therefore $\Vert X_t^d\Vert _{{\mathscr {F}}_0}$ is controlled overall as before (by an exponential, after considering the stochastic integral, etc) and we go back to (3.58) to now control $\displaystyle \int _0^t \Vert X_s^d\Vert _{{\mathscr {F}}_1}^2 ds$. Note that these calculations are in some sense similar in spirit with those from the previous case, although $C(\theta , \alpha _2)$ in

3.63 $$\begin{aligned} \begin{aligned} d\Vert X_t^d\Vert _{{\mathscr {F}}_0}^2 - C(\theta , \alpha _1)\Vert X_t^d\Vert _{{\mathscr {F}}_1}^2dt \le G(\Vert X_t^d\Vert _{{\mathscr {F}}_0})dt + \tilde{B}(X_t^d)dW_t \end{aligned} \end{aligned}$$

is now positive, but the role played by the η-viscous term in the previous case is now played by the term $\frac{\epsilon }{2}\langle N\rangle _t$ which was added ’artificially’ through the stochastic noise.

**Case III:** The diffusion coefficient is given by

3.64 $$\begin{aligned} B_t^d(X_t^d) = \theta \Vert X_t^d\Vert _{{\mathscr {F}}_0}^{\alpha _0}X_t^d. \end{aligned}$$

Then

3.65 $$\begin{aligned}  &   \begin{aligned} \Vert B_t^d(X_t^d)\Vert _{L_2({\mathscr {U}}, {\mathscr {F}}_1)}^2&= \theta ^2\Vert X_t^d\Vert _{{\mathscr {F}}_0}^{2\alpha _0}\Vert X_t^d\Vert _{{\mathscr {F}}_1}^2 \le \theta ^2\Vert X_t^d\Vert _{{\mathscr {F}}_1}^{2\alpha _0+2} \end{aligned} \end{aligned}$$

3.66 $$\begin{aligned}  &   \begin{aligned} \tilde{B}_t^d(X_t^d)^2&:=(2\langle X_t^d, B^d(X_t^d)\rangle _{{\mathscr {F}}_1})^2 = (2\theta \Vert X_t^d\Vert _{{\mathscr {F}}_0}^{\alpha _0}\Vert X_t^d\Vert _{{\mathscr {F}}_1}^2)^2 \\&= 4\theta ^2\Vert X_t^d\Vert _{{\mathscr {F}}_0}^{2\alpha _0}\Vert X_t^d\Vert _{{\mathscr {F}}_1}^4 \le 4\theta ^2\Vert X_t^d\Vert _{{\mathscr {F}}_1}^{2\alpha _0+4} \end{aligned} \end{aligned}$$

and

3.67 $$\begin{aligned} \begin{aligned} \tilde{A}_t^d(X^d)&=2{}_{\mathscr {D}}\langle X_t^d, A_t^d(X_t^d)\rangle _{{\mathscr {G}}} + \Vert B_t(X_t^d)\Vert _{L_2({\mathscr {U}}, {\mathscr {F}}_1)}^2 \\&= 2\langle X_t^d, A_t^d(X_t^d)\rangle _{{\mathscr {F}}_1} + \theta ^2\Vert X_t^d\Vert _{{\mathscr {F}}_0}^{2\alpha _0}\Vert X_t^d\Vert _{{\mathscr {F}}_1}^2\\&\le 2C_1 \Vert X_t^d\Vert _{{\mathscr {F}}_0}^{\gamma _{13}}\Vert X_t^d\Vert _{{\mathscr {F}}_1}^2+ \theta ^2\Vert X_t^d\Vert _{{\mathscr {F}}_0}^{2\alpha _0}\Vert X_t^d\Vert _{{\mathscr {F}}_1}^2 \\&\le 2C_1 \Vert X_t^d\Vert _{{\mathscr {F}}_0}^{\gamma _{13}}\Vert X_t^d\Vert _{{\mathscr {F}}_1}^2+ \theta ^2\Vert X_t^d\Vert _{{\mathscr {F}}_1}^{2\alpha _0+2}. \end{aligned} \end{aligned}$$

due to conditions (2.2) and (2.222.23). Then (3.29) becomes

3.68 $$\begin{aligned}&d \log \left( C + \Vert X_t^d\Vert _{{\mathscr {F}}_1}^2 \right) = \frac{1}{C+ \Vert X_t^d\Vert _{{\mathscr {F}}_1}^2}\left( \tilde{A}_t^d(X_t^d)dt + \tilde{B}_t^d(X_t^d)dW_t^d \right) \nonumber \\&\qquad - \frac{1}{2(C+ \Vert X_t^d\Vert _{{\mathscr {F}}_1}^2)^2}\tilde{B}_t^d(X_t^d)^2dt \nonumber \\&\quad = \frac{1}{C+\Vert X_t^d\Vert _{{\mathscr {F}}_1}^2}\tilde{A}_t^d(X_t^d)dt - \frac{1-\epsilon }{2(C+ \Vert X_t^d\Vert _{{\mathscr {F}}_1}^2)^2}\tilde{B}_t ^d(X_t^d)^2dt \nonumber \\&\qquad + \frac{1}{C+\Vert X_t^d\Vert _{{\mathscr {F}}_1}^2}\tilde{B}_t^d(X_t^d)dW_t^d - \frac{\epsilon }{2(C+\Vert X_t^d\Vert _{{\mathscr {F}}_1}^2)^2}\tilde{B}_t^d(X_t^d)^2dt\nonumber \\&\quad =\frac{1}{\left( C+\Vert X_t^d\Vert _{{\mathscr {F}}_1}^2\right) ^2}\left( (C+\Vert X_t^d\Vert _{{\mathscr {F}}_1}^2)\tilde{A}_t^d(X_t^d)-\frac{1-\epsilon }{2}\tilde{B}_t ^d(X_t^d)^2 \right) dt \nonumber \\&\qquad + dM_t - \frac{\epsilon }{2}d\langle M \rangle _t \nonumber \\&\quad \le \frac{1}{\left( C+\Vert X_t^d\Vert _{{\mathscr {F}}_1}^2\right) ^2} \left( (C+\Vert X_t^d\Vert _{{\mathscr {F}}_1}^2) (2C_1 \Vert X_t^d\Vert _{{\mathscr {F}}_0}^{\gamma _{13}}\Vert X_t^d\Vert _{{\mathscr {F}}_1}^2 \right. \nonumber \\&\qquad \left. + \theta ^2\Vert X_t^d\Vert _{{\mathscr {F}}_0}^{2\alpha _0}\Vert X_t^d\Vert _{{\mathscr {F}}_1}^2) - \frac{1-\epsilon }{2}4\theta ^2\Vert X_t^d\Vert _{{\mathscr {F}}_0}^{2\alpha _0}\Vert X_t^d\Vert _{{\mathscr {F}}_1}^4\right) dt + dM_t - \frac{\epsilon }{2}d\langle M \rangle _t \end{aligned}$$

3.69 $$\begin{aligned}&\quad = \frac{1}{\left( C+\Vert X_t^d\Vert _{{\mathscr {F}}_1}^2\right) ^2} \left( \tilde{C}_1\Vert X_t^d\Vert _{{\mathscr {F}}_0}^{\gamma _{13}}\Vert X_t^d\Vert _{{\mathscr {F}}_1}^2 + 2C_1\Vert X_t^d\Vert _{{\mathscr {F}}_0}^{\gamma _{13}}\Vert X_t^d\Vert _{{\mathscr {F}}_1}^4\right. \nonumber \\&\qquad \left. + \theta ^2\Vert X_t^d\Vert _{{\mathscr {F}}_0}^{2\alpha _0}\Vert X_t^d\Vert _{{\mathscr {F}}_1}^2 - 2(1-\epsilon )\theta ^2\Vert X_t^d\Vert _{{\mathscr {F}}_0}^{2\alpha _0}\Vert X_t^d\Vert _{{\mathscr {F}}_1}^4\right) dt + dM_t - \frac{\epsilon }{2}d\langle M \rangle _t \nonumber \\&\quad \le \frac{\Vert X_t^d\Vert _{{\mathscr {F}}_1}^2\Vert X_t^d\Vert _{{\mathscr {F}}_0}^{2\alpha _0}}{\left( C+\Vert X_t^d\Vert _{{\mathscr {F}}_1}^2\right) ^2} \left( (\tilde{C}_1 + \theta ^2) + (2C_1 - 2(1-\epsilon )\theta ^2)\Vert X_t^d\Vert _{{\mathscr {F}}_1}^2\right) \nonumber \\&\qquad + dM_t - \frac{\epsilon }{2}d\langle M \rangle _t \end{aligned}$$

and we choose θ and $\epsilon \in (0,1)$ such that $2C_1 - 2(1-\epsilon )\theta ^2 < 0$. Also, $\alpha _0 =\gamma _{13}/2$.

#### Remark 12

The previous proposition provides sufficient control conditions in order to pass to the limit in the stochastic term in the case of a compressible viscous model and in the case of an incompressible model, as presented in this paper. However, in order for the approximating stochastic integral to converge in the compressible inviscid model case too, we need uniform control also in ${\mathscr {D}}$. This is dictated by the special form of the diffusion term which now contains the ${\mathscr {F}}_1$ norm of the solution.^4^ We prove that such a control holds ${\mathbb {P}}$ - a.s. in the following proposition.

#### Proposition 13

The approximating sequence $(X_t^d)_d$ is ${\mathbb {P}}$ - a.s. uniformly controlled in ${\mathscr {D}}$, that is, for any $\epsilon _2 >0$ there exists ${\mathcal {K}}_2 >0$ such that

3.70 $$\begin{aligned} \displaystyle \sup _d {\mathbb {P}}\left( \displaystyle \sup _t \Vert X_t^d\Vert _{{\mathscr {D}}}^2 \ge {\mathcal {K}}_2\right) \le \epsilon _2. \end{aligned}$$

#### Proof

The approximating equation

3.71 $$\begin{aligned} dX_t^d = A_t^d(X_t^d)dt + B_t^d(X_t^d)dW_t^d \end{aligned}$$

holds in ${\mathscr {D}}^d$ endowed with the scalar product inherited from ${\mathscr {D}}$. By the Itô formula

3.72 $$\begin{aligned} \begin{aligned}&d\varphi (\Vert X_t^d\Vert _{{\mathscr {D}}}) = \frac{\partial \varphi }{\partial x}(\Vert X_t^d\Vert _{{\mathscr {D}}})\tilde{A}^d(X_t^d)dt \\&\quad + \frac{\partial \varphi }{\partial x}\tilde{B}^d(\Vert X_t^d\Vert _{{\mathscr {D}}})dW_t^d + \frac{1}{2}\frac{\partial ^2\varphi }{\partial x^2}(\Vert X_t^d\Vert _{{\mathscr {D}}})(\tilde{B}^d(X_t^d))^2dt \end{aligned} \end{aligned}$$

in $({\mathscr {D}}^d, \langle \cdot , \cdot \rangle _{{\mathscr {D}}})$, and we take

3.73 $$\begin{aligned} \varphi (\Vert x\Vert _{{\mathscr {D}}}):= \log (\Vert x\Vert _{{\mathscr {D}}}^2). \end{aligned}$$

The uniform control holds by the same arguments as those used in the proof of Proposition 11, with the only difference that here we use condition (2.202.21). There is, however, a subtlety in condition (2.202.21): as opposed to conditions (2.16) and (2.222.23), in (2.202.21) we do not require existence of a functional space which is of higher order compared to ${\mathscr {D}}$, on the right hand side. This is possible only in ${\mathscr {D}}$ and the justification hinges upon the fact that ${\mathscr {D}}^d$ is a finite-dimensional space (so all norms are equivalent), and the Fatou lemma.

### Tightness

#### Proposition 14

The approximating sequence of solutions $(X_t^d)_d$ is tight in $C\left( [0, T],{\mathscr {G}} \right)$.

#### Proof

In order to prove the tightness result, we use Theorem 16 and Proposition 11. We first show that for every t∈[0,T], the laws of $(X_t^d)_d$ are tight in ${\mathscr {G}}$. Then we prove that the sequence $(X^{d})_{d=1}^{\infty }$ satisfies the Aldous condition 5.1.

To prove tightness in ${\mathscr {G}}$ of the laws of $\left( X_t^{d}\right) _{d=1}^{\infty }$ for every t∈[0,T], it suffices to show that that for any ϵ>0, there exists a compact sets $K_{\epsilon }\in {\mathscr {G}}$ such that

$$\begin{aligned} \sup _{d\ge 1}{\mathbb {P}}\left( X_t^{d} \in {\mathscr {G}}\backslash K_{\epsilon }\right) \le \epsilon . \end{aligned}$$

Following from Proposition 11, we deduce that there exists ${\mathcal {K}}_{\epsilon }$ such that

$$\begin{aligned} \sup _{d}{\mathbb {P}}\left( \Vert X_{t}^{d}\Vert _{{\mathscr {F}}_{0}}\ge {\mathcal {K}}_{\epsilon }\right) \le \epsilon _{1}. \end{aligned}$$

Let $K_{\epsilon }=\left\{ x\in {\mathscr {G}}|\Vert x\Vert _{{\mathscr {F}} _{0}}\le {\mathcal {K}}_{\epsilon }\right\} \subset {\mathscr {G}}$. Since ${\mathscr {F}}_{0}$ is compactly embedded in ${\mathscr {G}}$ it follows that the set $K_{\epsilon }$ is compact in ${\mathscr {G}}$ and

$$\begin{aligned} \sup _{d\ge 1}{\mathbb {P}}\left( X_t^{d}\in {\mathscr {G}}\backslash K_{\epsilon }\right) \le \sup _{d}{\mathbb {P}}\left( \Vert X_{t}^{d}\Vert _{{\mathscr {F}}_{0}}\ge {\mathcal {K}}_{\epsilon }\right) \le \epsilon _{1} \end{aligned}$$

as required. We show below that the equivalent of condition (5.1) holds, with $\rho =\Vert \cdot \Vert _{{\mathcal {G}}}$. Let $\tau ^d$ be a positive stopping time. We need to check that for any ϵ>0, there exists δ>0 such that for every sequence $(\tau _{d})_{d=1}^{\infty }$ of $\left( {\mathcal {F}}_{t}\right) _{t\ge 0^{-}}$ - stopping times with $\tau _{d}\le T$

$$\begin{aligned} \sup _{d\ge 1}\sup _{0\le t\le \delta }{\mathbb {P}}\left( \left| \left| X_{\tau _{d}+t}^{d} -X_{\tau _{d}}^{d} \right| \right| _{{\mathscr {G}}}\ge \eta \right) \le \epsilon . \end{aligned}$$

We have

$$\begin{aligned} \Vert X_{\tau _{d}+t}^{d}-X_{\tau _{d}}^{d}\Vert _{{\mathscr {G}}}^{2}\le &   2\left\| \int _{\tau _{d}}^{\tau _{d}+t}A_{s}^{d}(X_{s}^{d})ds\right\| _{{\mathscr {G}}}^{2}+2 \left\| \int _{\tau _{d}}^{\tau _{d}+t}B_{s}^{d}(X_{s}^{d})dW_{s}\right\| _{{\mathscr {G}}}^{2} \\\le &   2t \int _{\tau _{d}}^{\tau _{d}+t}\left\| A_{s}^{d}(X_{s}^{d})\right\| _{{\mathscr {G}}}^{2}ds + 2\left\| \int _{\tau _{d}}^{\tau _{d}+t}B_{s}^{d}(X_{s}^{d})dW_{s}\right\| _{{\mathscr {G}}}^{2} \\\le &   2\delta \int _{\tau _{d}}^{\tau _{d}+t}\left\| A_{s}^{d}(X_{s}^{d})\right\| _{{\mathscr {G}}}^{2}ds + 2 \left\| \int _{\tau _{d}}^{\tau _{d}+t}B_{s}^{d}(X_{s}^{d})dW_{s}\right\| _{{\mathscr {G}}}^{2}. \end{aligned}$$

We prove first that for any $\epsilon ,\eta >0,$ there exists δ>0 such that

$$\begin{aligned} \sup _{d\ge 1}\sup _{0\le t\le \delta }{\mathbb {P}}\left( \delta \int _{\tau _{d}}^{\tau _{d}+t}\left\| A_{s}^{d}(X_{s}^{d})\right\| _{{\mathscr {G}}}^{2}ds\ge \eta \right) \le \epsilon . \end{aligned}$$

We divide the argument into three cases:

**Case I: **We have that

$$\begin{aligned} \int _{\tau _{d}}^{\tau _{d}+t}\left\| A_{s}^{d}(X_{s}^{d})\right\| _{ {\mathscr {G}}}^{2}ds\le &   C\left( \int _{\tau _{d}}^{\tau _{d}+t}\left\| X_{s}^{d}\right\| _{{\mathscr {F}}_{0}}^{2\gamma }ds+C\int _{\tau _{d}}^{\tau _{d}+t}\left\| X_{s}^{d}\right\| _{{\mathscr {F}}_{1}}^{2}ds+1\right) \\\le &   C\left( T\sup _{s\in \left[ 0,T\right] }\left\| X_{s}^{d}\right\| _{{\mathscr {F}}_{0}}^{2\gamma }+\int _{0}^{T}\left\| X_{s}^{d}\right\| _{{\mathscr {F}}_{1}}^{2}ds+1\right) . \end{aligned}$$

From the boundedness result, we choose R>0 such that

$$\begin{aligned} {\mathbb {P}}\left( \sup _{s\in [0,T]}\Vert X_{s}^{d}\Vert _{ {\mathscr {F}}_{0}}\ge R \right)\le &   \frac{\epsilon }{2} \\ {\mathbb {P}}\left( \int _{0}^{T}\left\| X_{s}^{d}\right\| _{ {\mathscr {F}}_{1}}^{2}ds\ge R \right)\le &   \frac{\epsilon }{2}. \end{aligned}$$

It follows that

$$\begin{aligned} {\mathbb {P}}\left( \left\| \int _{\tau _{d}}^{\tau _{d}+t}A_{s}^{d}(X_{s}^{d})ds\right\| _{{\mathscr {G}}}^{2}\ge C\left( TR^{2\gamma }+R+1\right) \right) \le \epsilon . \end{aligned}$$

We choose δ such that

$$\begin{aligned} \delta \left( C\left( TR^{2\gamma }+R+1\right) \right) =\eta \Leftrightarrow \delta =\frac{\eta }{C\left( TR^{2\gamma }+R+1\right) } \end{aligned}$$

which gives us

$$\begin{aligned}  &   {\mathbb {P}}\left( \delta \int _{\tau _{d}}^{\tau _{d}+t}\left\| A_{s}^{d}(X_{s}^{d})\right\| _{{\mathscr {G}}}^{2}ds\ge \eta \right) \\  &   \quad = {\mathbb {P}}\left( \left\| \int _{\tau _{d}}^{\tau _{d}+t}A_{s}^{d}(X_{s}^{d})ds\right\| _{{\mathscr {G}}}^{2}\ge C\left( TR^{2\gamma }+R+1\right) \right) \le \epsilon . \end{aligned}$$

**Case II: **We have that

$$\begin{aligned} \int _{\tau _{d}}^{\tau _{d}+t}\left\| A_{s}^{d}(X_{s}^{d})\right\| _{ {\mathscr {G}}}^{2}ds\le C\left( T\sup _{s\in \left[ 0,T\right] }\left\| X_{s}^{d}\right\| _{{\mathscr {D}}}^{2\gamma }+1\right) \end{aligned}$$

and the argument follows similar to case **Case I**.

**Case III: **We have that

$$\begin{aligned} \int _{\tau _{d}}^{\tau _{d}+t}\left\| A_{s}^{d}(X_{s}^{d})\right\| _{ {\mathscr {G}}}^{2}ds\le C\left( T\sup _{s\in \left[ 0,T\right] }\left\| X_{s}^{d}\right\| _{{\mathscr {F}}_{1}}^{2\gamma }+1\right) \end{aligned}$$

and the argument follows similar to case **Case I**.

For the stochastic integral we use Proposition 18. Let

3.74 $$\begin{aligned} M_{\tau _d, \tau _{d+t}} := \displaystyle \int _{\tau _d}^{\tau _d+t} B_s^d(X_s^d)dW_s \quad \langle M \rangle _{\tau _d,\tau _d+t} = \displaystyle \int _{\tau _d}^{\tau _d+t} (B_s^d(X_s^d))^2ds. \end{aligned}$$

We prove that for any ϵ>0, there exists δ>0 such that for every sequence $\left( \tau _{d}\right) _{d=1}^{\infty }$ of $\left( {\mathcal {F}} _{t}\right) _{t\ge 0^{-}}$ - stopping times with $\tau _d\le T$

3.75 $$\begin{aligned} \sup _{d\ge 1}\sup _{0\le t\le \delta }{\mathbb {P}}\left( \left| \left| M_{\tau _{d},\tau _{d+t}}\right| \right| _{{\mathscr {G}} }\ge \eta \right) \le \epsilon . \end{aligned}$$

We use here Proposition 18 to deduce that for any η,n>0,

$$\begin{aligned} {\mathbb {P}}\left( \left| \left| M_{\tau _{d},\tau _{d+t}}\right| \right| _{{\mathscr {G}}}\ge \eta \right)\le &   \frac{n}{\eta ^{2}}+\mathbb { P}\left( \langle M\rangle _{\tau _{d},\tau _{d}+t}>n\right) \\\le &   \frac{n}{ \eta ^{2}}+{\mathbb {P}}\left( \delta \sup _{t\in \left[ 0,T\right] }\left| \left| B_{t}^{d}(X_{t}^{d})\right| \right| _{{\mathscr {G}} }^{2}>n\right) \end{aligned}$$

and if we choose $n=\eta ^{2}\sqrt{\delta }$ we get that

$$\begin{aligned} {\mathbb {P}}\left( \left| \left| M_{\tau _{d},\tau _{d+t}}\right| \right| _{{\mathscr {G}}}\ge \eta \right) \le \sqrt{\delta }+{\mathbb {P}} \left( \sup _{t\in \left[ 0,T\right] }\left| \left| B_{t}^{d}(X_{t}^{d})\right| \right| _{{\mathscr {G}}}^{2}>\frac{\eta ^{2}}{\sqrt{\delta }}\right) . \end{aligned}$$

**Case I and III:** Here we assume that $B_{t}(X_{t})=\theta \Vert X_{t}\Vert _{{\mathscr {F}}_{0}}^{\alpha _{0}}X_{t}$. It follows that

$$\begin{aligned} \sup _{t\in \left[ 0,T\right] }\left| \left| B_{t}^{d}(X_{t}^{d})\right| \right| _{{\mathscr {G}}}^{2}=\theta ^{2}\sup _{t\in \left[ 0,T\right] }\Vert X_{t}^d\Vert _{{\mathscr {F}} _{0}}^{2\alpha _{0}}\left| \left| X_{t}^{d}\right| \right| _{{\mathscr {G}}}^{2}\le \theta ^{2}\sup _{t\in \left[ 0,T\right] }\Vert X_{t}\Vert _{{\mathscr {F}}_{0}}^{2\alpha _{0}+2} \end{aligned}$$

hence

$$\begin{aligned} {\mathbb {P}}\left( \left| \left| M_{\tau _{d},\tau _{d+t}}\right| \right| _{{\mathscr {G}}}\ge \eta \right) \le \sqrt{\delta }+{\mathbb {P}} \left( \sup _{t\in \left[ 0,T\right] }\Vert X_{t}\Vert _{{\mathscr {F}} _{0}}>\left( \frac{\eta ^{2}}{\sqrt{\delta }\frac{\eta ^{2}}{\sqrt{\delta }}} \right) ^{\frac{1}{2\alpha _{0}+2}}\right) \end{aligned}$$

and the result follows as above, by choosing $\delta \le \left( \frac{ \epsilon }{2}\right) ^{2}$ *and* such that

$$\begin{aligned} {\mathbb {P}}\left( \sup _{t\in \left[ 0,T\right] }\Vert X_{t}\Vert _{{\mathscr {F}} _{0}}^{2\alpha _{0}+2}>\left( \frac{\eta ^{2}}{\sqrt{\delta }\frac{\eta ^{2} }{\sqrt{\delta }}}\right) ^{\frac{1}{2\alpha _{0}+2}}\right) \le \frac{ \epsilon }{2} \end{aligned}$$

again by using Proposition 11.

**Case II:** Here we assume that $B_{t}(X_{t})=\theta \Vert X_{t}\Vert _{{\mathscr {F}}_{1}}^{\alpha _{1}}X_{t}$. It follows that

$$\begin{aligned} \sup _{t\in \left[ 0,T\right] }\left| \left| B_{t}^{d}(X_{t}^{d})\right| \right| _{{\mathscr {G}}}^{2}=\theta ^{2}\sup _{t\in \left[ 0,T\right] }\Vert X_{t}\Vert _{{\mathscr {F}} _{1}}^{2\alpha _{1}}\left| \left| B_{t}^{d}(X_{t}^{d})\right| \right| _{{\mathscr {G}}}^{2}\le \theta ^{2}\sup _{t\in \left[ 0,T\right] }\Vert X_{t}\Vert _{{\mathscr {F}}_{1}}^{2\alpha _{1}+2}. \end{aligned}$$

The analysis is then identical with that of **Case I and III **by applying Proposition 13 instead of Proposition 11. The tightness of the sequence is now complete.

## Applications to models from stochastic fluid dynamics

We enumerate below several partial differential equations that can be treated using the methodology we propose in this paper. The models in the first section are treated as part of Case I, the models in the second section are treated as part of Case II, while the models in the third section are covered by the theory developed in Case III.

### Viscous compressible and incompressible models

Incompressible Navier Stokes equation in vorticity form on the 3D torus (${\mathbb {T}}^{3}$)

4.1 $$\begin{aligned} d_{t}\boldsymbol{\omega } +{\mathcal {L}}_{v}\boldsymbol{\omega } =\Delta \boldsymbol{\omega }. \end{aligned}$$

In this context ${\mathcal {L}}_{v}\boldsymbol{\omega }$ denotes the Lie derivative with respect to the velocity field *v*, that is

4.2 $$\begin{aligned} {\mathcal {L}}_{v}\boldsymbol{\omega } :=(v\cdot \nabla )\boldsymbol{\omega } -(\boldsymbol{\omega } \cdot \nabla )v=: \left[ \,v\,,\,\boldsymbol{\omega } \,\right] . \end{aligned}$$

The velocity field *v* can be obtained via the Biot-Savart operator which in general reconstructs a zero-mean and divergence-free vector field *u* from a divergence-free vector field $\boldsymbol{\omega }$ such that $\textrm{curl}u = \boldsymbol{\omega }$. On the torus it is given by $u=-\textrm{curl}\Delta ^{-1}\boldsymbol{\omega }$. Here ${\mathscr {G}}:=L^{2}\left( {\mathbb {T}}^{3};{\mathbb {R}}^{3}\right)$ is the space of square integrable zero-mean and divergence-free three dimensional vector fields, ${\mathscr {F}}_{0}:=W^{2,2}\left( {\mathbb {T}}^{3};{\mathbb {R}}^{3}\right) ,{\mathscr {F}}_{1}:=W^{3,2}\left( {\mathbb {T}}^{3};{\mathbb {R}}^{3}\right) ,$ ${\mathscr {D}} :=W^{4,2}\left( {\mathbb {T}}^{3};{\mathbb {R}}^{3}\right) .$ The operator *A* is given by

$$\begin{aligned} A\left( \boldsymbol{\omega } \right) :=-\left[ \,v \,,\,\boldsymbol{\omega } \,\right] +\Delta \boldsymbol{\omega }. \end{aligned}$$

In this case, one can check that for $\boldsymbol{\omega } \in {\mathscr {D}}:=W^{4,2}\left( {\mathbb {T}}^{3};{\mathbb {R}}^{3}\right)$ there exists $\alpha ,\beta >0$ such that

$$\begin{aligned} \left\langle \boldsymbol{\omega } ,A\left( \boldsymbol{\omega } \right) \right\rangle _{W^{2,2}\left( {\mathbb {T}}^{3};{\mathbb {R}}^{3}\right) }\le \alpha \left| \left| \boldsymbol{\omega } \right| \right| _{W^{2,2}\left( {\mathbb {T}}^{3};{\mathbb {R}}^{3}\right) }^{6}-\beta \left| \left| \boldsymbol{\omega } \right| \right| _{W^{3,2}\left( {\mathbb {T}}^{3};{\mathbb {R}}^{3}\right) }^{2}. \end{aligned}$$

Moreover

$$\begin{aligned} \left| \left| A\left( \boldsymbol{\omega } \right) \right| \right| _{L^{2}\left( {\mathbb {T}}^{3};{\mathbb {R}}^{3}\right) }\le c\left( \left| \left| \boldsymbol{\omega } \right| \right| _{W^{2,2}\left( {\mathbb {T}}^{3};{\mathbb {R}}^{3}\right) }^{\frac{3}{2} }+\left| \left| \boldsymbol{\omega } \right| \right| _{W^{2,2}\left( {\mathbb {T}}^{3};{\mathbb {R}}^{3}\right) }\right) \end{aligned}$$

which implies that *A* is bounded on the compact sets $K = \left\{ \Vert \boldsymbol{\omega } \Vert _{W^{2,2}\left( {\mathbb {T}}^{3};{\mathbb {R}} ^{3}\right) }\le R\right\} \in L_{\sigma }^{2}\left( {\mathbb {T}}^{3};\mathbb { R}^{3}\right)$(this is required for tightness). Also the interpolation property holds for $L^{2}\left( {\mathbb {T}}^{3};{\mathbb {R}} ^{3}\right) ,$ $W^{2,2}\left( {\mathbb {T}}^{3};{\mathbb {R}}^{3}\right)$ and $W^{3,2}\left( {\mathbb {T}}^{3};{\mathbb {R}}^{3}\right)$.

**Viscous Rotating Shallow Water Model (2D)**

Let $u=(u^{1},u^{2})$ be the horizontal fluid velocity of the shallow water model, defined on the two-dimensional torus ${\mathbb {T}}^{2}$, and *h* be the thickness of the fluid column. Let also $v:=\epsilon u+{\mathcal {R}}$, with $curl\ {\mathcal {R}}=f\hat{z}$, and ${\mathcal {R}}$ corresponds to the vector potential for the (divergence-free) rotation rate about the vertical direction, and it is chosen here such that $\nabla {\mathcal {R}}=0$. We will denote by $a:=\left( v,h\right)$ the solution of the viscous rotating shallow water (RSW) system

$$\begin{aligned} {\textrm{d}}_{\textrm{t}}a_{t}=F\left( a_{t}\right) , \end{aligned}$$

where $$F\left( a_{t}\right) $$ denotes

$$\begin{aligned} F\left( \begin{array}{c} v \\ h \end{array} \right) =\left( \begin{array}{c} -u\cdot \nabla v-f\hat{z}\times u-\nabla p+\gamma \Delta v_{t} \\ -\nabla \cdot (hu)+\eta \Delta h_{t} \end{array} \right) \end{aligned}$$

and

- ϵ is the Rossby number, a dimensionless number which describes the effects of rotation on the fluid flow: a small Rossby number (ϵ<<1) suggests that the rotation dominates over the advective terms; it can be expressed as $\epsilon =\frac{U}{fL}$ where *U* is a typical scale for horizontal speed and *L* is a typical length scale.
- *f* is the Coriolis parameter, $f=2\Theta \sin \varphi$ where Θ is the rotation rate of the Earth and φ is the latitude; $f\hat{z} \times u=(-fu^{2},fu^{1})$, where $\hat{z}$ is a unit vector pointing away form the centre of the Earth. For the analytical analysis we sometimes assume *f* to be constant.
- $p:=\frac{h-b}{\epsilon {\mathscr {F}}_0}$, ∇p is the pressure term, *b* is the bottom topography function.
- ${\mathscr {F}}$ is the Froude number, a dimensionless number which relates to the stratification of the flow. It can be expressed as ${\mathscr {F}}=\frac{U}{NH}$ where *H* is the typical vertical scale and *N* is the buoyancy frequency.
- $\gamma , \eta$ are viscosity coefficients.

Here ${\mathscr {G}}:=L^{2}\left( {\mathbb {T}}^{2};{\mathbb {R}}^{2}\right)$ is the space of square integrable two dimensional vector fields, ${\mathscr {F}}_{0}:=W^{1,2}\left( {\mathbb {T}}^{2};{\mathbb {R}}^{2}\right) ,{\mathscr {F}}_{1}:=W^{2,2}\left( {\mathbb {T}}^{2};{\mathbb {R}}^{2}\right) ,$ ${\mathscr {D}}:=W^{3,2}\left( {\mathbb {T}}^{2};{\mathbb {R}}^{2}\right) .$ Then one can show that

4.3 $$\begin{aligned} \left\langle a,F\left( a\right) \right\rangle _{W^{1,2}\left( {\mathbb {T}}^{2}; {\mathbb {R}}^{2}\right) }\le C\left| \left| a\right| \right| _{W^{1,2}\left( {\mathbb {T}}^{2};{\mathbb {R}}^{2}\right) }^{6}-\epsilon \left| \left| a\right| \right| _{W^{2,2}\left( {\mathbb {T}} ^{2};{\mathbb {R}}^{2}\right) }^{2}. \end{aligned}$$

Moreover

$$\begin{aligned} \left| \left| F\left( a\right) \right| \right| _{L^{2}\left( {\mathbb {T}}^{2};{\mathbb {R}}^{2}\right) }\le c\left( \left| \left| a\right| \right| _{W^{1,2}\left( {\mathbb {T}}^{2};{\mathbb {R}} ^{2}\right) }^{2}+\left| \left| a\right| \right| _{W^{2,2}\left( {\mathbb {T}}^{2};{\mathbb {R}}^{2}\right) }+1\right) . \end{aligned}$$

### Inviscid compressible models

**Burgers’ equation** on the 2D torus (${\mathbb {T}}^2$)

4.4a $$\begin{aligned} d_{t}u+u\cdot \nabla u=0. \end{aligned}$$

In this case, the operator *A* is given by

$$\begin{aligned} A\left( u\right) :=-u\cdot \nabla u \end{aligned}$$

and ${\mathscr {G}}:=L^{2}\left( {\mathbb {T}}^{3};{\mathbb {R}}^{3}\right)$, ${\mathscr {F}}_{0}:=W^{1,2}\left( {\mathbb {T}}^{2};{\mathbb {R}}^{2}\right) ,{\mathscr {F}}_{1}:=W^{3,2}\left( {\mathbb {T}}^{2};{\mathbb {R}}^{2}\right)$ ${\mathscr {D}}:=W^{4,2}\left( {\mathbb {T}}^{2};{\mathbb {R}}^{2}\right) .$ We have

$$\begin{aligned} \left\langle u,A\left( u\right) \right\rangle _{W^{1,2}\left( {\mathbb {T}}^{2};{\mathbb {R}}^{2}\right) }\le &   C \left| \left| u\right| \right| _{W^{3,2}\left( {\mathbb {T}}^{2};{\mathbb {R}} ^{2}\right) }\left| \left| u\right| \right| _{W^{1,2}\left( {\mathbb {T}}^{2};{\mathbb {R}}^{2}\right) }^{2} \\ \left| \left| A\left( u\right) \right| \right| _{L^{2}\left( {\mathbb {T}}^{2};{\mathbb {R}}^{2}\right) }\le &   \left| \left| u\right| \right| _{W^{1,2}\left( {\mathbb {T}}^{2}; {\mathbb {R}}^{2}\right) }^{2} \\ \left\langle u,A\left( u\right) \right\rangle _{W^{4,2}\left( {\mathbb {T}}^{2};{\mathbb {R}}^{2}\right) }\le &   \left| \left| u\right| \right| _{W^{3,2}\left( {\mathbb {T}}^{2};{\mathbb {R}} ^{2}\right) }\left| \left| u\right| \right| _{W^{4,2}\left( {\mathbb {T}}^{2};{\mathbb {R}}^{2}\right) }^{2}. \end{aligned}$$

Note that (2.16) can be obtained from the first inequality by a direct application of Young’s inequality.

**Inviscid Rotating Shallow Water Model (2D)**

The rotating shallow water model (RSW) is as above, except the fact that there is no viscosity, that is $F(a_t)$ is replaced by $\bar{F}(a_t)$ and we have

$$\begin{aligned} {\textrm{d}}_{\textrm{t}}a_{t}=\bar{F}\left( a_{t}\right) , \end{aligned}$$

where

$$\begin{aligned} \bar{F}:=\left( \begin{array}{c} v \\ h \end{array} \right) =\left( \begin{array}{c} -u\cdot \nabla v-f\hat{z}\times u-\nabla p \\ -\nabla \cdot (hu) \end{array} \right) \end{aligned}$$

and ${\mathscr {G}}:=L^{2}\left( {\mathbb {T}}^{2};{\mathbb {R}}^{2}\right)$, ${\mathscr {F}}_{0}:=W^{1,2}\left( {\mathbb {T}}^{2};{\mathbb {R}}^{2}\right) ,{\mathscr {F}}_{1}:=W^{3,2}\left( {\mathbb {T}}^{2};{\mathbb {R}}^{2}\right)$ ${\mathscr {D}}:=W^{4,2}\left( {\mathbb {T}}^{2};{\mathbb {R}}^{2}\right)$ with similar inequalities as above.

### Inviscid incompressible models

**Incompressible Euler equation in vorticity form** on the 3D torus

4.5 $$\begin{aligned} d_{t}\boldsymbol{\omega } +{\mathcal {L}}_{v}\boldsymbol{\omega } =0 . \end{aligned}$$

Here ${\mathscr {G}}:=L^{2}\left( {\mathbb {T}}^{3};{\mathbb {R}} ^{3}\right)$, ${\mathscr {F}}_{0}:=W^{\frac{3}{2}+\epsilon ,2}\left( {\mathbb {T}}^{3};{\mathbb {R}}^{3}\right) ,{\mathscr {F}}_{1}:=W^{3,2}\left( {\mathbb {T}}^{3};{\mathbb {R}}^{3}\right) ,$ ${\mathscr {D}} :=W^{4,2}\left( {\mathbb {T}}^{3};{\mathbb {R}}^{3}\right) .$ In this case, one can check that for $\boldsymbol{\omega } \in {\mathscr {D}}:=W^{4,2}\left( {\mathbb {T}}^{3};{\mathbb {R}}^{3}\right)$

$$\begin{aligned} \left\langle \boldsymbol{\omega } ,A\left( \boldsymbol{\omega } \right) \right\rangle _{W^{2,2}\left( {\mathbb {T}}^{3};{\mathbb {R}}^{3}\right) }\le \alpha \left| \left| \boldsymbol{\omega } \right| \right| _{W^{\frac{3}{2} +\epsilon ,2}\left( {\mathbb {T}}^{3};{\mathbb {R}}^{3}\right) }\left| \left| \boldsymbol{\omega } \right| \right| _{W^{2,2}\left( \mathbb {T }^{3};{\mathbb {R}}^{3}\right) }^{2}. \end{aligned}$$

Moreover

$$\begin{aligned} \left| \left| A\left( \boldsymbol{\omega } \right) \right| \right| _{L^{2}\left( {\mathbb {T}}^{3};{\mathbb {R}}^{3}\right) }\le c\left| \left| \boldsymbol{\omega } \right| \right| _{W^{2,2}\left( {\mathbb {T}}^{3};{\mathbb {R}}^{3}\right) }^{\frac{3}{2}} \end{aligned}$$

which implies that *A* is bounded on compact sets $K = \left\{ \Vert \boldsymbol{\omega } \Vert _{W^{2,2}\left( {\mathbb {T}}^{3};{\mathbb {R}} ^{3}\right) }\le R\right\} \in L^{2}\left( {\mathbb {T}}^{3};\mathbb { R}^{3}\right)$. Also the interpolation property holds for $L^{2}\left( {\mathbb {T}}^{3};{\mathbb {R}}^{3}\right) ,$ $W^{\frac{3}{2} +\epsilon ,2}\left( {\mathbb {T}}^{3};{\mathbb {R}}^{3}\right)$ and $W^{2,2}\left( {\mathbb {T}}^{3};{\mathbb {R}}^{3}\right)$.

In general, global existence for the type of models presented in this section can be shown also in smoother spaces of the form $W^{k,2}({\mathbb {T}}^2,{\mathbb {R}}^2)$ with k≥2 (see e.g. [16, 17]). This is useful for numerical purposes.

## Appendix

**Aldous’ criterion for tightness**

Let $({\mathbb {S}}, \rho )$ be a separable and complete metric space. Let ${\mathcal {D}}(0, T; {\mathbb {S}})$ denote the set of ${\mathbb {S}}$-valued càdlàg functions defined on [0, *T*].

### Definition 15

A sequence $\left\{ X_n\right\} _{n=1}^{\infty }$ of ${\mathcal {D}}(0, T; {\mathbb {S}})$-valued random variables is said to satisfy the Aldous condition if and only if $\forall \epsilon>0, \forall \eta>0, \exists \delta >0$ such that for every sequence $\left\{ \tau _n\right\} _{n=1}^{\infty }$ of $\left( {\mathcal {F}}_t\right) _{t \ge 0^{-}}$ - stopping times with $\tau _n \le T$ we have

5.1 $$\begin{aligned} \sup _{n \ge 1} \sup _{0 \le t \le \delta } {\mathbb {P}}\left( \rho \left( X_n\left( \tau _n+t\right) , X_n\left( \tau _n\right) \right) \ge \eta \right) \le \epsilon . \end{aligned}$$

We can easily formulate a sufficient condition for (5.1) using Markov’s inequality. Suppose that there exist constants $\alpha , \beta , C>0$ such that for every sequence $\left\{ \tau _n\right\} _{n=1}^{\infty }$ of ${\mathcal {F}}_t$ - stopping times with $\tau _n \le T$ we have

5.2 $$\begin{aligned} \sup _{n \ge 1} {\mathbb {E}}\left( \rho \left( X_n\left( \tau _n+t\right) , X_n\left( \tau _n\right) \right) ^\alpha \right) \le C t^\beta \end{aligned}$$

for every t≥0. Then $\left\{ X_n\right\} _{n=1}^{\infty }$ satisfies the Aldous condition (5.1).

A sufficient condition for tightness of probability measures on ${\mathcal {D}}(0, T; {\mathbb {S}})$:

### Theorem 16

Let $({\mathbb {S}}, \rho )$ be a complete, separable metric space and let $\left\{ X_n\right\} _{n=1}^{\infty }$ be a sequence of ${\mathcal {D}}(0, T; {\mathbb {S}})$-valued random variables. If for every t∈[0,T] the laws of $\left\{ X_n(t)\right\} _{n=1}^{\infty }$ are tight on ${\mathbb {S}}$ and if condition (5.2) holds, then the laws of $\left\{ X_n\right\} _{n=1}^{\infty }$ are tight on ${\mathcal {D}}(0, T; {\mathbb {S}})$.

### Proposition 17

([23]) If *Y* is a continuous local martingale which vanishes at 0, then

5.3 $$\begin{aligned} {\mathbb {P}}\left( \displaystyle \sup _{t}{Y}_t \ge x, \quad \langle Y\rangle _t \le y \right) \le \frac{1}{e^{-x^2/2y}}. \end{aligned}$$

### Proposition 18

(Lemma 2.5 in [12]) Let *K*, *H* be separable Hilbert spaces, *Q* a trace-class operator on *K* and $\Phi \in L_2\left( K_Q, H\right)$, $K_Q=Q^{1/2}K$. Then for arbitrary δ>0 and n>0,

5.4 $$\begin{aligned} {\mathbb {P}}\left( \sup _{t \le T}\left\| \int _0^t \Phi (s) d W_s\right\| _H>\delta \right) \le \frac{n}{\delta ^2}+{\mathbb {P}}\left( \int _0^T\Vert \Phi (s)\Vert _{L_2\left( K_Q, H\right) }^2 d s>n\right) . \end{aligned}$$

## Data Availability

No datasets were generated or analysed during the current study.

## Footnotes

1 So $$T_d{\mathscr {G}} = {\mathscr {D}}^d$$.

2 For further standard details related to such a projection see e.g. [24] Section 4.1.

3 Note that $\tilde{C}_2$ can be equal to $2C_2, C_2$, or something else, depending on the model. See Section 4 for more explicit details.

4 Note that the control for $\displaystyle \sup _{t\in [0,T]}\Vert X_t^d\Vert _{{\mathscr {F}}_0}$ and $\displaystyle \int _0^T\Vert X_t^d\Vert _{{\mathscr {F}}_1}dt$ still holds as before also in the compressible inviscid case, but these two conditions are not sufficient.
